# Conversational Agents Supporting Self-Management in People With a Chronic Disease: Systematic Review

**DOI:** 10.2196/72309

**Published:** 2025-08-26

**Authors:** Tessa F Peerbolte, Rozanne JA van Diggelen, Pieter van den Haak, Kim Geurts, Luc JW Evers, Bastiaan R Bloem, Nienke M de Vries, Sanne W van den Berg

**Affiliations:** 1 Department of Neurology, Center of Expertise for Parkinson and Movement Disorders Donders Institute for Brain, Cognition and Behavior Radboud University Medical Center Nijmegen The Netherlands; 2 Department Human Movement Sciences University Medical Center Groningen Groningen The Netherlands

**Keywords:** conversational agents, chatbots, self-management, behavior change techniques, patient education, chronic disease, delivery of health care, artificial intelligence, AI

## Abstract

**Background:**

Conversational agents (CAs) are increasingly used as a promising tool for scalable, accessible, and personalized self-management support of people with a chronic disease. Studies of CAs for self-management of chronic disease operate within a multidisciplinary domain: self-management originates from (behavioral) psychology and CAs stem from intervention technology, while diseases are typically studied within the biomedical context. To ensure their effectiveness, structured evaluations and descriptions of the interventions, integrating biomedical, behavioral, and technological perspectives, are essential.

**Objective:**

We aimed to examine the design and evaluation of CAs for self-management support of chronic diseases, focusing on their characteristics, integration of behavioral change techniques, and evaluation methods. The findings will guide future research and inform intervention design.

**Methods:**

We conducted a systematic search in the PubMed and Embase databases to identify studies that investigated CAs for chronic disease self-management, published from January 1, 2018, to April 15, 2024. Full-text journal articles, published in English, studying the efficacy or effectiveness of a CA in the context of self-management for chronic diseases in adults were included. Data extraction was guided by conceptual frameworks to ensure comprehensive reporting of intervention and methodologies: the behavioral intervention technology model and the CONSORT-EHEALTH (Consolidated Standards of Reporting Trials of Electronic and Mobile Health Applications and Online Telehealth) checklist. Risk of bias was assessed using the Risk of Bias 2 tool and the Risk of Bias in Non-randomized Studies-of Interventions (ROBINS-I) tool (version 2).

**Results:**

In total, 25 studies were included, primarily focusing on text-based, rule-based CAs delivered via a mobile apps. The chronic diseases predominantly targeted were diabetes and cancer. Commonly identified clusters of behavior change techniques were “shaping knowledge,” “feedback and monitoring,” “natural consequences,” and “associations.” However, reporting of behavior change techniques and their delivery was lacking, and intervention descriptions were limited. Studies were mostly in the early phase, with a great variety in intervention descriptions, study methods, and outcome measures.

**Conclusions:**

Advancing the field of CA-based interventions requires transparent intervention descriptions, rigorous methodologies, consistent use of validated scales, standardized taxonomy, and reporting aligned with standardized frameworks. Enhanced integration of artificial intelligence–driven personalization and a focus on implementation in health care settings are critical for future research.

## Introduction

### Background

Chronic diseases are the leading cause of mortality and morbidity and impose a significant global burden on patients, their families, and the health care system [1,2]. Tackling these challenges requires strategies to empower patients in self-managing their disease effectively. Self-management is a promising strategy to treat chronic diseases, enabling patients to actively identify and solve illness-related problems to prevent complications or reduce disability [3]. Self-management interventions improve health behavior, health outcomes, and quality of life (QoL) and reduce health care use [4,5]. Conversational agents (CAs), web- or app-based computer programs that engage in 2-way interactions by simulating humanlike conversations, have emerged as a promising tool for self-management support [6].

Self-management refers to an individual’s ability to manage symptoms, treatments, physical and psychosocial consequences, and lifestyle changes [7]. Self-management involves role, medical, and emotional management tasks and requires problem-solving, decision-making, resource use, patient–health care provider partnership formation, action planning, and self-tailoring skills [5]. To deploy these skills, patients should be knowledgeable about their illness, symptoms, and available treatments, including self-care options, to make appropriate decisions about their disease management [5]. CAs facilitate self-management by providing knowledge (eg, answering questions about the disease [8]), promoting self-management skills (eg, decision-making based on self-monitoring data), and assisting with self-management tasks (eg, by offering emotional support [8]). CAs can serve as multicomponent technology-based behavior change interventions by incorporating behavior change techniques (BCTs). BCTs are observable, replicable, and irreducible components designed to alter or redirect causal processes that regulate behavior [9]. For example, a CA designed to support mental well-being in people with chronic headaches contains the BCT “goals and planning,” and the technology delivers this technique to the user by assisting them to plan a mindfulness-based activity during the next week [10]. Early evidence highlights that CAs hold significant promises to support self-management of chronic diseases, showing acceptability, usability, and potential efficacy for improving self-management [6,11,12].

CAs are present in diverse forms and functionalities, ranging from systems with pre-programmed responses to more sophisticated embodied artificial intelligence (AI) agents. The field of health care CAs is rapidly evolving, especially with the new developments in AI. A key development is the emergence of large language models (LLMs), deep learning models designed to process and generate natural language text. LLMs excel in generating meaningful humanlike creativity, reasoning, and contextually appropriate language [8,13], allowing users of CAs to converse with the CA as they would with other humans [8]. Well-known examples of CAs that use LLMs are ChatGPT and, more recently, DeepSeek. The rise of AI may further enhance the potential of CAs as self-management interventions.

CAs for chronic disease self-management operate in a multidisciplinary context, integrating intervention technology, (behavioral) psychology, and medical or biomedical sciences. Therefore, rigorous evaluation of their effectiveness in chronic disease management is complex, and a consensus on the best approach is lacking [6,11,12,14]. A comprehensive perspective that incorporates frameworks from all relevant disciplines is essential for effective evaluation [15]. Also, previous reviews typically focused on specific subsets of CAs—such as text-based [6], voice-based [16], AI-driven [11,12,14] or embodied [17] agents—resulting in an incomplete understanding of intervention content and design.

### This Study

A previous scoping review [18] outlined various types of CAs used in chronic condition management, along with their characteristics and study designs. Expanding on this work, we adopt a multidisciplinary perspective by integrating frameworks from diverse domains, including the CONSORT-EHEALTH (Consolidated Standards of Reporting Trials of Electronic and Mobile Health Applications and Online Telehealth) checklist, the behavioral intervention technology (BIT) model, and BCTs. Through this synthesis, we provide a comprehensive overview of CAs developed for chronic disease self-management support and their characteristics and evaluation approaches. The findings offer practical insights for developers and researchers, ultimately contributing to the design of robust, evidence-based CA health interventions.

## Methods

We adhered to the PRISMA (Preferred Reporting Items for Systematic Reviews and Meta-Analyses) guidelines for conducting systematic reviews (Multimedia Appendix 1) [19]. Our protocol was not registered.

### Search Strategy

The search was performed in the PubMed and Embase databases, covering papers published between January 1, 2018, and April 15, 2024. We prioritized sensitivity over specificity to ensure that all potentially relevant studies were included. Therefore, we based our search string on synonyms for CA and derived no search terms from “self-management” and “chronic disease,” as these are broad concepts that are hard to capture within a set of terms. Our approach resulted in the following search string for PubMed: “Conversational robot*” [tiab] OR “conversational agent*” [tiab] OR “chat bot*” [tiab] OR “chat agent*” [tiab] OR “conversationalagent*” [tiab] OR “chatbot*” [tiab] OR “chatterbot*” [tiab] OR “virtual agent*” [tiab] OR “virtual robot*” [tiab] OR “relational agent*” [tiab] OR “conversational assistant*” [tiab] OR “speech assistant*” [tiab] OR “chat assistant*” [tiab] OR “virtual assistant*” [tiab] OR “chatassistant*” [tiab] OR “AI agent*” [tiab] OR “dialogue system*” [tiab] OR “voice assistant*” [tiab]” OR “ai agent*”[Title/Abstract] OR “dialog system*”[Title/Abstract] OR “voice assistant*”[Title/Abstract]. The search string was adjusted for use in Embase. The initial search was performed in PubMed on May 1, 2023, and an updated search in PubMed was performed on April 15, 2024. To improve the comprehensiveness of our search, we added "AI agent", "dialogue system", and "voice assistant" to our search string in PubMed on March 25, 2025, and repeated our full search in the Embase database on March 27, 2025.

### Study Selection Criteria

Inclusion criteria were (1) full-text journal articles; (2) published in English; (3) from 2018 to April 15 2024, to account for rapid technological advancements; and (4) included a CA in; (5) the context of self-management for; (6) chronic diseases in; (7) adults; and (8) reporting primary research findings evaluating efficacy, including pre- and postuse measures.

Chronic disease was defined, according to the Centers for Disease Control and Prevention, as “conditions that last 1 year or more and require ongoing medical attention or limit activities of daily living or both.” [20] Our selection on self-management was guided by the definition of Barlow et al [7] as an individual’s ability to manage symptoms, treatments, physical and psychosocial consequences, and lifestyle changes. We added the criterion that CAs should at least offer information or education, as these components are essential for developing the knowledge and skills necessary to manage chronic diseases effectively [5]. Studies investigating one-time use of a CA, virtual reality agents, mental health, addiction, or neurodiversity were considered out of the scope of this review and were therefore excluded.

### Study Selection

One researcher (TFP) screened the studies’ eligibility based on title and abstract, rating them as “potentially relevant” or “not relevant.” Doubtful cases were reviewed by 2 other researchers (SWvdB and NMdV). Subsequently, the full text of potentially relevant articles was assessed by one researcher (TFP). Uncertainties were discussed with the 2 other researchers (SWvdB and NMdV).

### Data Extraction and Synthesis

#### Overview

One researcher (TFP) extracted the data in a standardized form using the abstract, main text, supplemental material, and previous publications describing the CA under study if referred to by the authors. To generate a comprehensive overview of the CA description and study methods, we used the BIT model [10] and CONSORT-EHEALTH checklist [21]. The BIT model defines the conceptual and technological architecture of BITs. CAs for self-management can be considered as BITs, as they function as mobile health or eHealth interventions that support users in changing behaviors and cognitions. The model includes reporting on the intervention aims (the “why”), behavior change strategies (the conceptual “how”), by what elements these strategies are implemented in technology (the “what”), when an intervention component is delivered (the “when”), and intervention characteristics to meet the user’s needs and preferences (the technical “how”). The CONSORT-EHEALTH checklist was developed to improve reporting of eHealth trials and serves as a basis for evaluating the validity and applicability of eHealth trials.

#### Characteristics of the CA

We described CAs using the “technical how” component of the BIT model and additional characteristics based on the CONSORT (Consolidated Standards of Reporting Trials) checklist, extracting data on mode of delivery, access, embodiment status, conversational techniques, in- and output modalities, and recommended dose.

Mode of delivery refers to how the CA is delivered to the users, such as via an app. Method of access reflects how users access the mode of delivery, such as by downloading the app through a link provided via WhatsApp. We specified conversational techniques, encompassing input processing and response generation methods, as rule-based or AI-driven. We considered the conversational techniques AI-driven if the input was processed using AI (eg, using natural language processing to understand user input, context, and recognize patterns) or AI was used to respond in a humanlike conversational manner (eg, using LLMs) or both. In contrast, rule-based systems do not adapt or learn from user input and provide preprogrammed responses. Input and output modalities refer to how users communicate with the CA (input modality, eg, text) and how the CA communicates with the user (output modality, eg, speech). Recommended dose refers to the planned or recommended amount (eg, duration or content completion, such as one lesson) or frequency (eg, once a day) of exposure to the intervention for optimal effectiveness.

Finally, we described any cointerventions, which are elements of the intervention that are not part of the CA itself.

#### Integration of BCTs

On the basis of the BIT model, we extracted the clinical aim (eg, reducing blood sugar), BCT clusters (eg, feedback and monitoring), and BCT delivery by technology (eg, providing self-care guidance based on monitored blood sugar levels). A BCT is an “observable, replicable, and irreducible component of an intervention designed to alter or redirect causal processes that regulate behaviour” and BCTs are categorized into clusters [9]. If not reported, 3 researchers (TFP, RJAvD, and SWvdB) identified BCT clusters based on intervention descriptions and BCT taxonomy [9] in consensus meetings.

#### Evaluation Methods

To describe the evaluation method, we extracted study characteristics (the study’s aim, design, follow-up period, location, participant eligibility criteria related to technical use and skills, intervention and control conditions, and demographics [age, gender, education, and technical skills and use]) and outcome measures. To ensure a comprehensive overview, we reported outcome measures in the following categories: clinical, feasibility, and acceptability outcomes. We adhered to the authors’ classification when provided and self-categorized otherwise. Clinical outcomes included disease-specific, psychological, behavioral, and knowledge and skills measures. Feasibility included recruitment, enrollment, attrition, accrual, exclusion, and retention rates, and outcomes based on use data. We categorized user experience measures under acceptability.

#### Primary or Coprimary Study Results

The focus of this review is not to draw conclusions on the effectiveness, feasibility, or acceptability of CAs for chronic disease self-management support. However, to get an overview of whether CAs seem to work for the self-management of chronic diseases, we chose to report the results of the primary or coprimary outcomes.

### Study Risk of Bias Assessment

We only reported primary study outcomes in this review. Risk of bias for primary outcomes that evaluated efficacy or effectiveness was assessed using the Cochrane Risk of Bias in Non-randomized Studies-of Intervention (ROBINS-I) tool (version 2 [22]) for nonrandomized studies and the Risk of Bias 2 tool [23] for randomized controlled trials. One reviewer (TFP) individually assessed the quality of eligible studies; the assessments were discussed with SWvdB and NMdV.

## Results

### Overview

**Figure 1:**
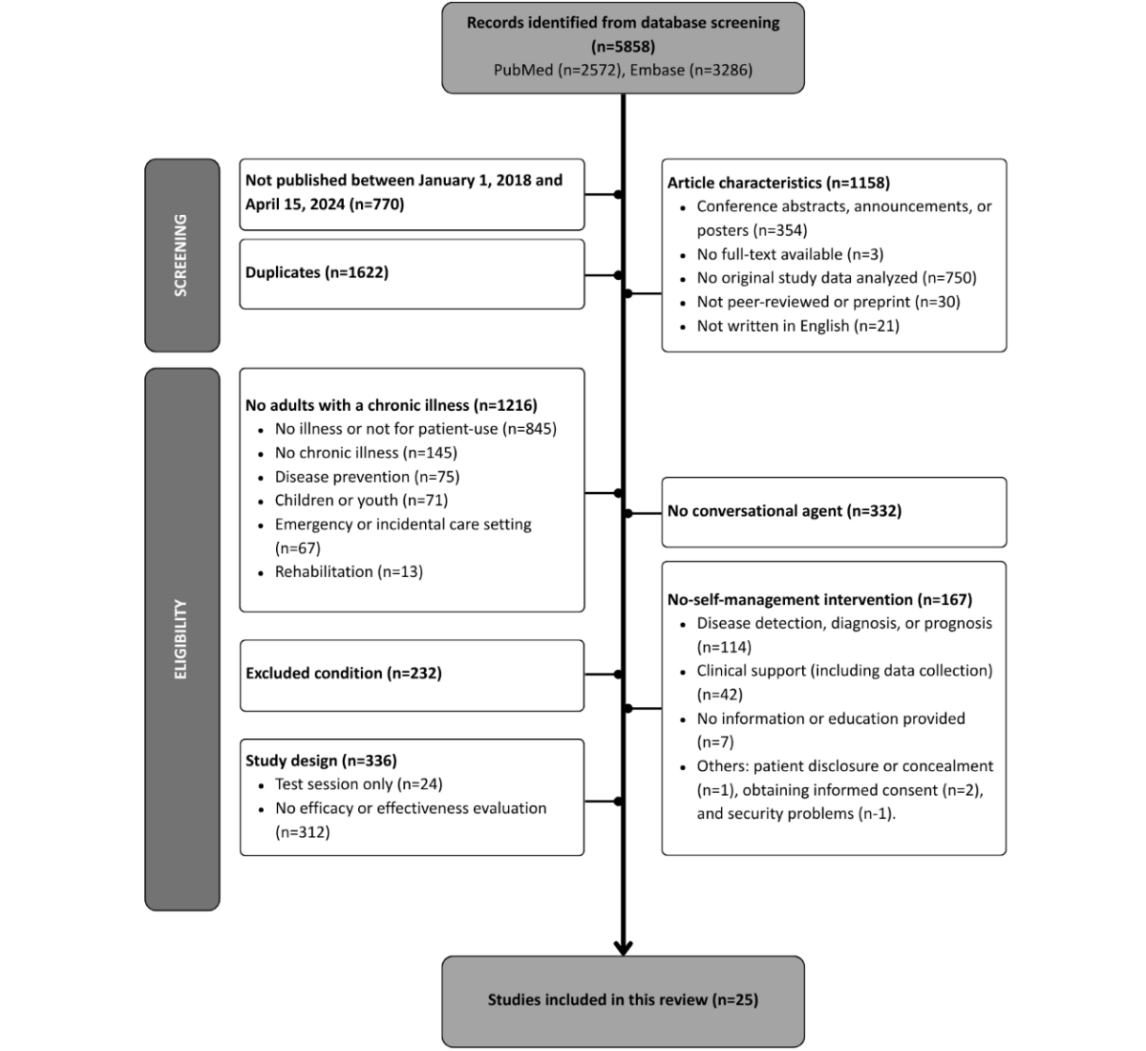
Flowchart for the article screening process.

Our initial search in the PubMed database on May 1, 2023, yielded 1215 results. A subsequent search on April 15, 2024, resulted in 1114 additional hits. The expanded search of the additional terms yielded 243 hits, and the Embase database search yielded 3286 hits. In total, 5858 articles were screened. After screening, we excluded 5497 articles based on title and abstract. After full-text screening, 25 articles were included. Figure 1 illustrates the inclusion process details.

### Characteristics of the CAs

Table 1 summarizes the characteristics of 23 unique CAs included in this study. Two studies evaluated nurse Addressing Metastatic Individuals Everyday [24,25] and Wysa [26,27]. “Chatbot” was the predominant used term (13/23, 57%) [28-40]. Most CAs (18/23, 78%) operated through mobile apps [26,27,29-34,36-39,41-46] as stand-alone apps or integrated into existing apps such as Telegram [36] or Signal [44]. The majority were unembodied agents (14/23, 61%) that communicated through text [26-33,35,36,40,44,46-48]. Embodied agents used speech and animated behaviors [24,25,41-43,45]. CAs were often supplemented with multimedia, such as audio recordings or images. Embodiment status of 4 CAs [34,37-39] and output modalities for 4 CAs [34,37,39,40] were not reported.

Input modalities varied, including natural language text, voice, predefined choices, and combinations. Rule-based conversational techniques were most common (10/23, 43%) [24,25,29,30,33,36,41,42,44-46,48]. Some CAs (4/23, 17%) solely relied on AI-driven conversational techniques [26-28,34,39] while others (4/23) used a combination of AI-driven and rule-based techniques [24,25,32,40,43,47]. Some descriptions of conversational techniques or input modalities were incomplete [31,32,34,35,37-39]. The recommended dose varied from daily interactions to use frequency and duration based on the users’ preference [26,27,32,34,37-39,46] but sometimes lacked (5/23, 22%). Most CAs (15/23, 65%) were part of broader co-interventions, including additional app functionalities, physical and digital information sources, physical self-monitoring devices, and human involvement [24,25,31,32,34-41,43,45,46,48]. Human roles encompassed monitoring symptoms and activity, conducting weekly check-ins, and updating individualized goals [24,25]; monitoring of disabling conditions and addressing users’ questions [31]; responding to questions the chatbot could not answer and delivering contextually tailored messages [32]; reviewing patient data, providing personalized feedback, and answering queries [34]; and managing technical alerts [43]. Clinicians were also involved in responding to flagged participant responses [35,36].

**Table 1:** Characteristics of the conversational agents (CAs).

Name of CA, terminology used, and study		Instantiation—how (technical)					
		Device, mode of delivery, and method of access	Embodiment	Conversational techniques	In- and output modalities	Recommended dose	Description of cointervention
**Atrial fibrillation**							
	Tanya, relational agent, Guhl et al [41]	Mobile app installed on smartphone	Yes	Rule-based	Input: menu-based selections Output: spoken, written, and visuala	Daily interactions	Self-monitoring device “AliveCor’s Kardia” to measure heart rate.
**Cancer**							
	Chemofree, chatbot, Tawfik et al [28]	Devices with the Android operating system, accessed via a link received on WhatsApp.	No	AI^b^-based	Input: written (natural language text and keywords) Output: written	Continuous access with user-initiated conversations.	N/A^c^
	Vivibot, chatbot, Greer et al [29]	Device not reported, but accessible via Facebook messenger (web- and app-based). Log-in account required.	No	Rule-based	Input: written (natural language text, predefined options, and emojis). Output: written and multimedia content.	Structured daily interactions for 28 d.	N/A
	Nurse AMIE^d^, virtual assistant, Caru et al [24], Schmitz et al [25], and Qiu et al [49]	Tablet (Echo Show study device), via Amazon Alexa with a voice command, “Alexa, open nurse AMIE.”	Yes	AI-driven (voice) and rule-based (touch)	Input: spoken and touch. Output: spoken, written, and visual.	Daily use, 1 lesson a day for 28 d	Coupled devices: Amazon Echo Show, pedometer, and resistance bands. Participant binder including (palliative) care team information, instructions for Amazon Eecho Show, nurse AMIE and pedometer, step log sheets, instructions on the exercises, and nutrition information. Study facilitator: monitors user activity and reported symptoms. Weekly calls asked users how they were feeling, reviewed elevated symptom responses, and asked if they were having any difficulties or questions about the program. Reviewed lifestyle activities the user was using to manage their symptoms and updated individualized step goals based on the steps in the user’s record.
	*Nameless,* Chatbot, Gomaa et al [30]	Mobile app (text messaging) on smartphone, the app is not stored on a device.	No	Rule-based	Input: written (predefined topic keywords) Output: written	3 interactions a week	N/A
	*Nameless,* chatbot, Huang et al [31]	Device not reported. Access through a QR code leading to the Facebook messenger interface (web- and app-based); log-in account required.	No	*Unclear*	Input: written (natural language text or predefined answer options is unclear). Output: written	Daily interactions	Outcomes of assessments done by the chatbot were monitored by a cancer manager, if a disabling condition (CTCAE^e^ grade >3) was registered or if the chatbot was unable to respond to a user’s question, the cancer manager contacted the user to initiate a phone or online discussion and to provide any direct help if needed.
	*Nameless, chatbot or CA,* Albino de Queiroz et al [40]	Via the Facebook messenger app and webpage on a notebook, smartphone, or tablet.	No	Rule-based and AI-driven	Input: text and click-based Output: unclear	Not reported	Wearable devices collecting physical activity data, which were integrated into the chatbot.
***Chronic disease***							
	*Nameless,* interactive reminder-based app, Fang et al [42]	Mobile app preinstalled with user information entered on an iPad study device	Yes	Rule-based	Input: click-based Output: spoken and visual	On the basis of users’ medication intake schedule	N/A
**CKD^f^**							
	*CIM-SHE*^*p*^*program,* chatbot, Chen et al [32]	Via a preinstalled mobile app instant messaging on smartphone	No	Rule based, *possibly* with AI-driven components.	Input: written (predefined options, keywords, or natural language text is unclear) Output: written	Not reported	Head nurse of nephrology that answers the keyword queries that showed no match in the chatbot. The participant is notified of this with the message “Please wait; we will reply as soon as possible.” The nurse also receives a notification once a question comes in. The nurse collects health information associated with CKD care and broadcasts it on the Line app to prompt users with contextually tailored messages to increase their engagement.
***Chronic pain***							
	Wysa, CA, Meheli et al [27] and Cheng et al [26]	Device not specified. Via a mobile app that runs on iOS and Android devices and can be voluntarily downloaded via a publicly available link for free.	No	AI-based	Input: written Output: written	Not reported by Meheli et al [27] and ≥3 interactions a week reported by Cheng et al [39].	N/A
	Selma, chatbot, Hauser-Ulrich et al [33]	Mobile app downloaded via the project webpage or directly via the App Store or Google Play Store on an iOS or Android smartphone	No	Rule-based	Input: predefined answer options (click-based), written (natural language text for entering nickname and numerical to enter age) Output: written and multimedia	Daily interactions for 8 wk	N/A
***Diabetes***							
	*Nameless,* chatbot, Krishnakumar et al [34]	Mobile app downloaded from the Google Play store using a unique link sent to the user via SMS or text message on an Android smartphone	Not reported	Unclear; reports on AI-powered decision support but does not describe conversational techniques.	Input: entered self-care and diabetes management data, context of the previous clinical, lifestyle, and behavioral data (interaction input and output modality not specified).	Not reported	Chatbot embedded in the Wellthy CARE digital therapeutic app that delivers a structured self-management program based on a digital persuasion model focusing on improving motivation, reducing difficulty, and delivering appropriate triggers. Consists of coaching, messaging with a health care coach (virtual diabetes educator), self-log self-care data and diabetes management information, a learning library, lesson plans, a logbook, a gamification element, and AI-powered decision support. A glucose measurement device is included.
	Elena, virtual assistant, Roca et al [44] and Roca et al [50]	Mobile app signal downloaded on smartphone and user registration by the nurse during medical appointment	No	Rule-based	Input: written (predefined choices or indicated by a number) Output: written and pictures.	Multiple times daily	N/A
	Laura, CA, Gong et al [43]	Mobile app on a smart device with an operating system of at least iOS 8 for Apple or OS 4.2 for Android	Yes	Rule-based (touch-based selections) and AI-based (interactive voice recognition)	Input: spoken or touch-based selections Output: spoken and visual	Scheduled weekly appointments with CA	Embedded in the MDC^h^ program, also encourages regularly accessing the user guide and the MDC website and joining discussion forums on diabetes self-management (posted by the program coordinator). The program coordinator assisted users in dealing with system-generated technical alerts by communicating with the users, the general practitioner, and the technology company.
	*Nameless, CA*, Bruijnes et al [47]	Not reported	No	AI-driven (free-text) and rule-based (predefined choices).	Input: Written (free-text) and predefined choices (button-based). Output: written	Weekly interactions	N/A
	*Nameless,* chatbot, Nassar et al [35]	Web app via mobile devices (desktop or tablet) the app does not require a download on device.	No	Rule-based	Input: written (natural language text) and predefined choices (button-based). Output: written and multimedia.	1st mo: weekly and >1 mo: based on user preference	A dashboard tracked enrollment data, participant feedback, and the identified need for escalation via flags based on participant responses.
***HIV***							
	*Nameless,* realistic talking human avatar, Dworkin et al [45]	The mobile app was downloaded from the study laptop to the participants’ smartphones by the study team.	Yes	Rule-based	Input: predefined choices (touch-based). Output: spoken, visual, multimedia, and text.	Daily interactions	Embedded in the My personal health guide app that includes (1) enabling direct phone calls to health care providers; (2) sending reminders; (3) facilitating monitoring of self-entered data; (4) providing feedback on the monitored data (including warnings and rewarding images and autotune); (5) giving information on medication, how to use the app, and app goals and theory behind it; (6) enabling users to send bug reports; (7) options for customization; and (8) protecting users’ privacy with password log-in, default log-out, and disguisable tap screen.
***Hypertension***							
	Tensiobot, chatbot, Echeazarra et al [36]	The mobile app Telegram is downloaded and installed on a smartphone. All assisted by the nurse (including registration)	No	Rule-based	Input: predefined choices (text-, voice-, and button-based), numeric input. Output: written, links, and media objects	Interactions twice a day	Physicians receive notifications if BP^i^ is abnormal.
	*Nameless,* chatbot, Sakane et al [37]	Device not reported. Mobile app downloaded via Google Play or App Store.	Not reported	Unclearly described	Unclearly described	Approximately daily	Embedded in the KENPO app developed using self-monitoring and goal-setting theory. The app collects data from questionnaires, laboratory results, personal goals, and self-monitoring using coupled devices (data are actively measured and directly uploaded to the app). It also provides specific health guidance based on app data and personality traits.
***Irritable bowel syndrome***							
	Zemedy, chatbot, Hunt et al [38]	Mobile app downloaded via a link on an iOS or Android smartphone	No	Not reported	Input: written (unclear) Output: written and audio.	Approximately weekly	Delivered together with a toolkit that contains a health check, breathing, imagery, relaxation, hypnotherapy, and yoga.
***Kidney failure***							
	PD^j^-AI chatbot, chatbot, Cheng et al [39]	Mobile app LINE on a smart device	Not reported	AI-based	Not reported	Not reported	An administrator (nurse) interface via which they can (1) access the patient interface, allowing the nurse to monitor and interact with the content that patients are engaging with, and (2) track user interaction, for example, number of clicks.
***Overactive bladder***							
	CeCe, CA, Sheyn et al [48]	Web app on an iOS or Android smartphone; assistance from the website via a research coordinator	No	Rule-based	Input: click-based Output: written and audio and video instructions.	Interactions of 5-10 min daily for 8 wk	Documents with exercise instructions that could be downloaded via an email link. Diary function, sleep or wake cycle information and peeing or drinking, and a review function (contains recaps of lessons from the chats directing to links to PDFs).
***Primary headaches***							
	David or Sophie, CA, Ulrich et al [46]	The mobile app self-downloaded via the study website on an Android or iOS smartphone.	No	Rule-based	Input: predefined answer options and text Output: written media objects (videos and pictures).	As preferred, from daily to weekly	Part of the BalanceUP app (1) chat channel; (2) audio library (eg, relaxation, mindfulness, and imagination exercises); (3) illustrations; (4) working materials (eg, energy balance and coping circle); (5) video library (eg, animated psychoeducational videos); and (6) frequently asked questions about the study and BalanceUP app.

^a^Visual modes of communication include various channels of expression (eg, visual cues such as facial expressions and body movement).

^b^AI: artificial intelligence.

^c^N/A: not applicable.

^d^AMIE: Addressing Metastatic Individuals Everyday.

^e^CTCAE: Common Terminology Criteria for Adverse Events

^f^CKD: chronic kidney disease.

^g^CIM-SHE: Chat-based Instant Messaging Support Health Education.

^h^MDC: My Diabetes Coach.

^i^BP: blood pressure.

^j^PD: peritoneal dialysis.

### Integration of BCTs

On the basis of intervention descriptions, we identified BCT clusters and delivery methods (Table 2). All authors reported the aim of their CA—except for one [37]. The most frequently identified BCT clusters were “shaping knowledge” (17/23, 74% [24-26,29-33,35,36,38,39,41,44-48]), “feedback and monitoring” (15/23, 65% [24-26,29-31,33-35,37,40,41,43,46,48]), and “natural consequences” (13/23, 57% [24-26,30,33,35,38, 39,41,42,44-47]). “Shaping knowledge” equips individuals with practical knowledge (eg, how to perform a behavior) and cognitive strategies to adopt and sustain desired behaviors. This was delivered mostly by behavioral instructions, for example, how to perform relaxation [46]. “Feedback and monitoring” refer to strategies to monitor or self-monitor behavior and its outcomes and deliver feedback. Monitoring often involved (prompted) assessments of health outcomes [24,25,29-31,35] or behaviors [35,36,48]. Feedback included self-care recommendations based on the data. “Natural consequences” involves strategies that help to understand the relationship between behavior and its consequences. This was delivered through information about the necessity of self-care behaviors and cognitions [26,30,38,46] or medication intake [41,42,44]. “Shaping knowledge” was often combined with “feedback and monitoring,” providing personalized self-care guidance based on the user’s symptoms [24,25,30,31,47]. In addition, “associations” were often incorporated (14/23, 61% [24,29-31,33,34,36,38,40-42,44-46]), often with prompts to use the CA. “Social support” was delivered by facilitating contact with health care providers [35]; videos of fellow patients [29,45]; advice on and arranging social support and practical help from friends and relatives [46]; or being the actor that provides the social support [24,25,30,31,33,45,46].

**Table 2:** Technical integration of behavior change techniques (BCTs).

Study, why? (aim of the CA^a^), and how? (conceptual, eg, BCTs)					What? (ie the elements through which the BCTs are integrated in the technology)
**Atrial fibrillation**					
	**Guhl et al [41]**				
		**To improve medication adherence and health-related quality of life**			
			Natural consequences	Dialogue containing modules focusing on disease education, symptoms, and adherence.	
			Shaping knowledge	Dialogue providing instructions on how to use the Kardia device (heart rate monitoring device).	
			Feedback and monitoring	Monitoring (not further specified) Dialogue directing users to check the rhythm concomitant with reporting symptoms.	
			Goals and planning	Refers to previous content areas and obtain repeated assessments to follow for the resolution of reported problems.	
			Associations	Dialogue referring to Kardia to reinforce its use	
			No BCT; personalized experience	Uses the user’s name and appropriate time context (eg, good afternoon) and responds to the user’s conversational inputs from the current and prior conversations.	
**Cancer**					
	**Tawfik et al [28]**				
		**To improve the effectiveness of self-care behaviors and reduce the frequency, severity, and distress of chemotherapy side effects**			
			Shaping knowledge and natural consequences^b^	Respond to queries in natural language. Coupled to a knowledge base that was based on the most asked questions and American Cancer Society guidelines 2020. Users could select from a list of commonly experienced chemotherapy-related side effects, and the chatbot then provided a detailed answer.	
	**Greer et al [29]**				
		**To deliver cognitive and behavioral intervention to increase positive emotion**			
			Feedback and monitoring	Daily emotional ratings at the start of each session.	
			Shaping knowledge, repetition, and substitution	Conversational lessons for positive psychology skills based on stress and coping theory and broaden-and-build theory of positive emotion. Teaching lesson: introduces a skill to users and allows guided reflection. Practice lesson: practice the skills with a script that focuses on guided reflection.	
			No BCT; give space to vent	After the completion of the skill, the user was given an opportunity to “vent” or “free-write” what was on their mind.	
			Social support	Video of a cancer survivor (optional to view after completing a skill)	
			Associations	User receives a daily notification with a basic greeting (eg, “Hey, it’s me, your friendly neighborhood bot!”) which the user could respond to if they wanted to chat. Daily prompts for emotion ratings. Notification of the availability of the next lesson 24 h after completing the previous one.	
	**Schmitz et al [25] and Caru et al [24]**				
		**Schmit et al [25]: to record relevant symptoms (eg, the level of pain), provide customized interventions (based on metastasis location and symptoms), and deliver nutrition tips; Caru et al [24]: to enhance daily step counts in women with MBC^c^**			
			Associations	Unique greeting for everyday users to stimulate opening Nurse AMIE^d^. Unique farewells for each day of use.	
			Natural consequences	Daily nutrition tips and educational content related to coping with symptoms and nutritional advice are provided. These tips are presented through voice and visual means (eg, recipes and nutritional advice).	
			Social support	The voice interface includes cheerful greetings and conversational engagement, fostering a supportive dialogue that encourages users to interact with the system regularly. Empathic responses based on collected user data (eg, “I’m so glad you are feeling so well”)	
			Feedback and monitoring	Asks users about their symptom status and daily activity. Users can record data using a voice interface. Uploads data to investigator dashboard. Flagging: if symptom scores are above a threshold, CA (1) informs the user, “be sure to contact the clinical care team about your elevated symptoms.”, and (2) alerts the care team about elevated symptoms by email or phone call.	
			Feedback and monitoring, antecedents, and shaping knowledge^b^	Offers self-care interventions (soothing music, CBT^e^ lessons, guided relaxation, exercise videos [balance, strengthening, and stretching], or audio messages) based on symptom ratings or user choice.	
			Goal setting	Users provide feedback on previous interventions, which allows Nurse AMIE to adjust difficulty levels for future exercise recommendations, effectively helping users set achievable fitness goals.	
			No BCT; personalized experience	To provide an engaging and satisfying experience: dynamic content changes based on users’ behavior, preferences, and interests.	
	**Gomaa et al [30]**				
		**To optimize the care experience for patients with gastrointestinal cancer receiving chemotherapy, ultimately contributing to improved outcomes and enhanced patient well-being**			
			Associations, social support, natural consequences, and shaping knowledge^f^	Tailored information about chemotherapy-related side effects and strategies for preventing and managing side effects, recommendations for self-care strategies and lifestyle changes, emotional support, and guidance on effective communication with health care providers and family members. Delivered by proactive text messages.	
			Associations, shaping knowledge and natural consequences^b^, feedback and monitoring, and no BCT; personalized experience	CA prompts users to engage by (1) allowing users to request additional information, coping strategies, and evidence-based real-time recommendations to support their self-management tailored to individuals needs and symptoms. (2) Immediate self-care feedback based on reported symptoms to deliver timely and tailored support.	
			Associations and feedback and monitoring	Encourages users to regularly assess and report their symptoms. By prompting structured weekly assessments via text messages, where the user responds to specific questions about their symptoms.	
	**Huang et al [31]**				
		**To decrease ED** ^g^ **use and reduce unscheduled hospitalizations by collecting patient-reported symptoms during chemotherapy treatment and automatically alerting clinicians to severe or worsening symptoms**			
			Associations, feedback and monitoring, and shaping knowledge	Prompts the user to report their symptoms daily Delivers tailored instructions and suggestions about how to relieve their symptoms.	
			Social support	CA uses intelligence and empathy to engage with users.	
			Feedback and monitoring and shaping knowledge	CA starts with a greeting, an emotion assessment, and a quick follow-up response for mental health support to facilitate the conversation. CA follows up on patients’ daily conditions. Sequential question-and-answer for symptom assessment. If users experienced symptoms other than those listed, they could use the text message function to talk to the chatbot. Information extraction by medical entity recognition. Natural language generation of user responses based on predefined templates of health instructions. At enrollment, CA sends notifications regarding the users’ conditions. The users respond to the CA based on their symptoms. The CA sends prearranged suggestions about how to relieve these symptoms. If patients presented with disabling conditions (CTCAE^h^ grade >3), CA software was unable to respond to a patient’s questions.	
	**Albino de Queiroz et al [40]**				
		**To encourage patients to be more involved in their treatment, improve self-management, and report on their clinical condition**			
			Shaping knowledge^f^	The patient interacts passively and actively with the chatbot using cognitive behavioral therapy techniques. Addresses the flows of the most common symptoms and adverse effects related to chemotherapy, physical activity the patients perform, food consumption (according to the nutritionist’s guidelines), and questionnaires (quality of life of patients with cancer and colorectal).	
			Feedback and monitoring	Provides feedback to the patient based on the provided data and the interaction. Data are collected and integrated from medical records, self-reports, and wearable devices. Diagrams were developed to cover the chatbot’s symptoms and adverse effects, contemplating specific flows for each level of criticality of data reported by the patient.	
			Associations	Sends notifications in case of noninteraction.	
			No BCT, flagging system	Notifies the multidisciplinary team if deterioration of the patient’s clinical condition is identified.	
**Chronic disease**					
	**Fang et al [42]**				
		**To increase adherence and improve overall user satisfaction by simulating a human carer or physician through an avatar**			
			Associations	Verbal notifications are generated based on a predefined schedule (ie, when users need to take their supplements).	
			Natural consequences	Provides simple information related to the supplement the user takes with one click on the button.	
			No BCT; personalization	Reminders and information are tailored to individual user supplements and supplement intake schedules.	
**CKD^i^**					
	**Chen et al [32]**				
		**To improve participants’ health literacy related to communication ability and disease-specific knowledge**			
			Associations	Line push notifications prompt users about health information related to CKD.	
			Shaping knowledge	Users ask questions related to disease information by entering a keyword. For example, “high-phosphorus diet” could be entered to search for information about high-phosphorus foods. Presents several questions based on fuzzy comparison from which the user could choose and displays the corresponding answer. No matched questions and answers? “Please wait; we will reply as soon as possible.” The head nurse of a nephrology ward responds to these queries personally. As the manager of this chatbot, the nurse immediately receives notifications about queries requiring responses.	
			Reward and threat	Offers awards to enhance user engagement for (1) asking problem-discovery questions to participants who ask novel questions associated with their daily lives provoking creative and insightful discussions to solve problems, (2) the user who asks the most questions, and (3) the user who most often interacts with the chatbot system and promptly responds to Line push notifications.	
			Human involvement	CA asks after answering a question: “Are you satisfied with this answer?” If the participant responds “dissatisfied,” the manager would promptly improve the answer or clarify the context of the participants’ questions and provide modified responses to improve the intervention’s effectiveness.	
**Chronic pain**					
	**Meheli et al [27]**				
		**To promote mental health**			
			—^f^	Recommending evidence-based elements from CBT, behavioral reinforcement, and mindfulness, among others. The self-help practices and conversation-based tools and techniques provide support for challenges, including anxiety, sleep, low energy, motivation, loss, and pain.	
	**Cheng et al [39]**				
		**To deliver therapeutic content, including cognitive behavioral therapy, cognitive restructuring, motivational interviewing, mindfulness training, deep breathing techniques, and sleep meditations to collectively improve users’ behavioral activation, pain acceptance, and sleep quality**			
			Natural consequences	CA guides users through a week-by-week curriculum that uses behavioral activation and pain acceptance principles to encourage users to engage with things that bring joy, despite having pain.	
			Feedback and monitoring	Daily check-ins	
			Shaping knowledge and repetition and substitution^b^	Night sleep meditations	
			Monitoring feedback	Weekly progress reports and a progress road map	
			Reward and threat	Unlock premium reward tool packs by engaging with the weekly reports.	
	**Hauser-Ulrich et al [33]**				
		**To promote self-management of chronic pain**			
			Shaping knowledge	Participants learn about the utility of a pain diary and are instructed to apply it over the following 2 wk	
			Natural consequences and shaping knowledge^b^	Information delivered through daily messages regarding chronic pain (management) the importance of applying psychological therapy and diagnostics in pain management. Explains the relationship between chronic pain, thoughts, emotions, social impacts, and the process of chronification. Information delivered through dedicated modules (incl. exercise instructions) regarding dysfunctional cognitions, behavior, and emotions (eg, stress, fear of pain, and anxiety) and coping strategies (eg, activity, resources, mindfulness, and acceptance)	
			Feedback and monitoring	Provides information on psychoeducation based on the participants’ input regarding pain intensity and duration.	
			No BCT, personalized experience	Users can select 3 modules. The conversational path is determined based on screening questions and changes over time based on the user’s input about prior knowledge and interest.	
			No BCT, technical feature	Saves missed messages. Makes them accessible to the users. Ensures questionnaire completion by requiring each question displayed on the screen to be completed, otherwise, the user is unable to proceed.	
			Goals and planning, social support, and feedback and monitoring	Addresses accountability by referring to earlier tasks and activities, supporting activity and task completion, and sending motivational messages. Shows an overview of the intervention schedule and all unfinished modules.	
			Associations	Personalized text messages every other day (also shows as a notification). If CA sends a message and CA is closed, a sticky notification is generated (ie, the notification is displayed in the notification dashboard and acts as a reminder). Moreover, 48 h after the last notification, the next messaging sequence is started.	
			Social support	Imitates a real human chat-based conversation using emojis and some sense of humor. Shows social support by expressing sympathy and affective empathy and placing the emphasis on the user’s achieved tasks.	
**Diabetes**					
	**Krishnakumar et al [34]**				
		**To enhance multiple behavior patterns (self-monitoring of diet, exercise, weight, and blood glucose)**			
			Feedback and monitoring^f^	Gives automated personalized feedback delivered in real-time through a conversational experience. Provides educational, behavioral, and motivational messaging specific to entered data and in the context of previous clinical, lifestyle, and behavioral data.	
	**Roca et al [50]**				
		**To improve medication adherence**			
			Associations and repetition and substitution	Messages when it is time to take the medication (3 consecutive reminders every 10 min until response). The message includes a picture of the medication box.	
			No BCT; personalized experience and technical features	Shows (1) current medication and drug history and allows to add or delete medication and (2) medical appointments and allows them to add, modify, or delete medical appointments.	
			Goals and planning and monitoring and feedback	Shows medication intake summary and remaining medication to be taken during the day. Provides a weekly adherence summary.	
			Natural consequences	Shows pictures of the medication box and provides an information pamphlet.	
			Shaping knowledge	Lists tutorials that are available on YouTube that explain how to use the chatbot.	
			No BCT; persuasive feature in increasing CA use	Daily weather forecast	
	**Gong et al [43]**				
		**To provide more accessible and engaging self-management support, monitoring, and coaching**			
			Feedback and monitoring and shaping knowledge^f^	Education and counseling: delivers personalized support, monitoring, and motivational coaching through a series of modules (chosen by the user) covering blood glucose monitoring, healthy eating, physical activity, medication taking, and foot care. Includes tips on overcoming barriers.	
			No BCT; personalized experience	The number of appointments (with CA, weekly) per module depends on users’ responses during the interactions, enabling a high degree of program personalization.	
			Goals and planning	The user experience is personalized and tailored based on the clinical targets and glucose monitoring frequency recommendations provided by users’ GPs^j^.	
			Feedback and monitoring	Progress is reviewed based on monitoring data, and based on this, the chatbot delivers feedback.	
			—^f, k^	Short quiz	
	**Bruijnes et al [47]**				
		**To help people deal with social problems caused by diabetes**			
			No BCT	Starts every session explaining its goal for the current session.	
			Goals and planning	Defines the problem, that is, which social diabetes distress is most problematic for the user (how?).	
			Shaping knowledge and natural consequences^b^ and personalized experience	Presents strategies appropriate to deal with that specific distress (user-tailored advice).	
			Comparison of outcomes	Actively engage the user by asking them to come up with potential solutions to their problem, discussing the advantages and disadvantages of the appropriate solutions, and asking the user which solution they prefer.	
			Natural consequences	Presents the preferred strategy purpose in more detail to establish trust in the strategy and the agent.	
			Goals and planning	Reflect with the user on whether the strategy presented in the previous session worked and, if not, whether another strategy should be explored, or whether another distress should be addressed.	
	**Nassar et al [35]**				
		**To improve self-care confidence and to improve A_1C_^l^**			
			Feedback and monitoring	Assessment of knowledge and self-care confidence. Self-reporting of blood glucose data and medication-taking behaviors. Session content varies based on answers provided and patient knowledge assessments taken during the conversations. A traffic light pattern: “green” is used to identify “on track” patients, and “yellow” or “red” prompt further questions by the chat platform or drive an intervention by a clinician.	
			Natural consequences and shaping knowledge^b^	Education content: 30 s to 4-min microlearning videos and written diabetes self-management education and support content based on the Association of Diabetes Care & Education Specialists 7 self-care behaviors.	
			No BCT; patient-initiated use	On-demand sessions occur any time a patient clicks or reclicks on a notification and is not scheduled for a chat.	
			Associations and goals and planning	Fixed sessions based on users’ preference (notifications also on the day of the patient’s preference).	
			Social support	Allows users to reach out to clinical staff.	
			No BCT; flagging system	Escalation process in case of alarming signals: The care team is messaged during office hours; beyond office hours, the user is instructed to call the primary care provider or go to the emergency room if immediate help is required.	
**HIV**					
	**Dworkin et al [45]**				
		**To address adherence and retention in HIV care with the overarching goal of improving viral suppression**			
			Natural consequences and shaping knowledge^b^	Giving answers to educational questions containing rationales for healthy behavior (eg, What can happen if I get AIDS?)	
			Social support	Blends motivational statements into educational information; responses include motivation; relation (such as empathy, empowerment, or other dialogue that might elicit an emotional response); and interaction (eg, are you afraid to take your meds?).	
			Social support and comparison of outcomes	Offers motivational audio messages from other HIV-care stakeholders, for example, other young male individuals who are HIV positive and have sex with male individuals and authorities (eg, physicians) speaking, reflecting on their own experiences, and encouraging healthy behavior.	
			Shaping knowledge	Provides an audiovisual orientation to the home screen and its available functions.	
			Associations	When a user comes to the home tab or when the app is restarted, the agent asks the user, for example, “Do you want to hear what a friend of mine says?” and “Do you know what I think?” The goal is to make the app more interactive and less user-directed.	
			No BCT; personalized experience	Choose the agent’s appearance and select the user’s name (if it is among the 95 names that the agent can speak) to be used in greetings and responses.	
**Hypertension**					
	**Echeazarra et al [36]**				
		**To assist patients with high blood pressure in performing home blood pressure checks**			
			Feedback and monitoring, associations	Asks users to measure their blood pressure two consecutive times, twice a day. Indicates the interval between the 2 consecutive measurements. Asks users questions regarding the measurement outcomes in between the 2 consecutive blood pressure measures. Shows a line chart with the blood-pressure history after the 2 consecutive measurements. Asks to confirm (if so, CA notifies physician) or contradict (eg, due to typo; if so, CA asks to reenter the measurement) if a measurement is out of line. Asks user to perform a third measurement after 2 min, if 2 consecutive measures differ by >5 mm Hg.	
			Shaping knowledge and natural consequences^b^	Displays a helpful video on good blood measurement practice (including tips) and sends messages with tips related to good blood measurement practice. Once a day, the chatbot sends a message with other tips related to good blood pressure measurement practices.	
			No BCT; personalized experience and technical features	Knows a command to remind the patient of the scheduled time for the next medical appointment, allowing the patient to change or cancel it directly through a conversation with the chatbot.	
	**Sakane et al [37]**				
		**NR^m^**			
			Shaping knowledge and natural consequences^b^	Chatbot-supported health information on health and health behavior was selected from 392 quizzes based on app data that was provided to the participants.	
			Feedback and monitoring	Chatbot supported feedback on app data and signs of weight regain	
**IBS^n^**					
	**Hunt et al [38]**				
		**To treat irritable bowel syndrome with CBT**			
			Natural consequences^f^	Two modules of psychoeducation about the etiology of IBS and CBT’s effectiveness in treating it.	
			Antecedents and associations, natural consequences, repetition and substitution, and shaping knowledge	Eight modules teaching the users about various CBT strategies to mitigate the impact of IBS on daily life, including relaxation training, exercise, cognitive restructuring and decatastrophizing, exposure exercises to reduce avoidance, and behavioral experiments. Also encourages a healthy diet.	
			Associations	Prompts users to apply these strategies in their daily lives.	
			Antecedents	In case of acute pain or anxiety management, exercises help to directly mitigate distress and discomfort.	
**Kidney failure**					
	**Cheng et al [39]**				
		**To improve the self-care ability of patients undergoing peritoneal dialysis**			
			Shaping knowledge and natural consequences^b^	Peritoneal dialysis instructional videos with content on manual exchange, automated peritoneal dialysis operation, exit site dressing, shower protection, intraperitoneal heparin, and intraperitoneal antibiotics. Home peritoneal dialysis care with content on drainage and refill troubleshooting, contamination of the catheter or rupture, peritonitis, hemoperitoeal, catheter and tunnel infection, fluid and intake control, questions and answers, and peritonitis prediction. Peritoneal dialysis diet guide with content on protein intake, anemia diet, potassium-rich foods, phosphorus-containing foods, and preventing hyperlipidemia diet. Automated peritoneal dialysis guidance with content on automated peritoneal dialysis on-board and reception, automated peritoneal dialysis question and answer, common warnings, common mistakes, power failure, modem installation, and activation codes.	
			Association and goals and planning	Clinical reminders with content on peritonitis prevention, catheter self-care, nutritionist education, home self-injection teaching, instructional videos, and adequacy collection.	
			No BCT, technical feature	Hospital registration service with content on internet registration, medication inquiry, polymerase chain reaction reservation, and vaccine reservation.	
**Overactive bladder**					
	**Sheyn et al [48]**				
		**To provide first-line behavioral modification therapy for the treatment of overactive bladder**			
			Feedback and monitoring and goals and planning	CeCe collects patient information, such as a bladder diary, patient goals, and symptoms.	
			No BCT; personalized experience	Uses complex proprietary algorithms to assess and deliver personalized treatment.	
			Shaping knowledge and repetition and substitution^f^	Includes a variety of behavioral strategies, such as pelvic floor muscle exercises, bladder training, urge suppression, fluid management, and dietary modification.	
			Goals and planning^f^	Users can guide their own learning with review sessions and self-reflection exercises.	
**Primary headaches**					
	**Ulrich^o^ et al [46]**				
		**To improve mental well-being by promoting BCTs in behaviors, emotions, thoughts, and beliefs related to headaches while ensuring low-threshold access and scalability**			
			Goals and planning	Example: Agree on a mindfulness-based activity during the next week.	
			Feedback and monitoring	Example: Inform the participants of minutes of relaxation and imagination exercises performed so far.	
			Social support	Example: Constant encouragement by the coach for applied relaxation.	
			Shaping knowledge	Example: Elaborated instructions on how to perform relaxation.	
			Natural consequences	Example: Explain that taking medication more than 10 times per month can cause headaches.	
			Comparison of behavior	Example: By means of video, demonstrate to participants how to perform the imagination exercise “sensory isolation and stimulus shielding.”	
			Associations	Example: Send push notifications on participants’ smartphones to remind them to relax.	
			Repetition and substitution	Example: Encourage participants to practice relaxation techniques in noisy places as well; this will help them incorporate these techniques into their daily routines more easily, instead of just relying on quiet environments.	
			Comparison of outcomes	Example: Present information based on the latest scientific knowledge and research, in collaboration with experts, such as scientists, physicians, and psychologists, and following established guidelines.	
			Reward and threat	Example: Provision of a certificate only if the coaching was completed within a predefined number of days.	
			Regulation	Example: Explain the use of different medications and recommend taking antiemetics in combination with pain medication in case of a migraine.	
			Antecedents	Example: Advise preparing sports equipment the night before—to have it ready when leaving the house.	
			Identity	Example: Unconditional perseverance should be avoided at the onset of headaches. Taking a break should not be interpreted as a weakness.	
			Scheduled consequences	Example: Arrange with a person to reward themselves for being physically more active (eg, to use stairs).	
			Self-belief	Example: Tell the person that they can successfully adopt relaxation and imagination, even if it seems very hard to focus from time to time.	

^a^CA: conversational agent.

^b^Indistinguishable due to lack of details on the intervention.

^c^MBC: metastatic breast cancer.

^d^AMIE: Addressing Metastatic Individuals Everyday.

^e^CBT: cognitive behavioral therapy.

^f^Likely more BCT’s involved but due to lack of details on the intervention, these cannot be clearly identified.

^g^ED: emergency department.

^h^CTCAE: Common Terminology Criteria for Adverse Events.

^i^CKD: chronic kidney disease.

^j^GP: general practitioner.

^k^Not available.

^l^A_1C_: hemoglobin A_1c_.

^m^NR: not reported.

^n^IBS: inflammatory bowel disease.

^o^Described the integrated BCT with examples of how the BCT were integrated into the app. Only the clusters are reported in this table, with one example per category.

One study [46] integrated the BCTs into their intervention with explicit examples of delivery by technology. All others lacked explicit reporting on which and how BCTs were delivered. The details in which the CA was described varied. Ambiguous intervention descriptions hindered the classification of BCTs. As an example, “recommending evidence-based elements from cognitive behavioral therapy, behavioral reinforcement, and mindfulness, among others” [27] suggests the integration of many BCTs but does not allow us to identify which and how BCTs are delivered. The insufficient reporting on CA content made distinguishing “shaping knowledge” from “natural consequences” challenging [26,30,35,39,47]. For example, “providing information about medication” could refer to instructions on its use (“shaping knowledge”) or explaining the effect and importance of adherence (“natural consequences”).

### Evaluation Methods

#### Study Characteristics

In total, 24 unique studies were reviewed (Tables 3 and 4), as the findings of 1 study were published in 2 separate articles [24,25]. Most studies (11/24, 46%) were randomized controlled trials [24,25,28,29,33,36-38,41-43,46]. Most (14/24, 58%) were early-phase studies, such as pilot studies, initial trials, or exploratory studies [24,26,29,30,32,33,35,37,41,42,44,45,47,48]. Follow-up periods ranged from 3 weeks to 1 year. More than half of the studies (13/24, 54%) reported primary or coprimary outcomes [28,29,31,33-35,38,39,41,43,45,46,48]. Primary or coprimary outcomes were predominantly clinical outcomes, and user satisfaction was assessed as a primary outcome once [39]. One study [29] lacked reporting of which outcome was primary. Many studies (17/24, 71%) selected participants on technical skills (eg, ability to install apps or communicate online) [37,40,41], device access or possession [28-30,32,33,35,45,46,48], or both [34,36,38,44]. CAs were accessible to the participants primarily in addition to usual care, although this was not always specified. In one study [28], the CA replaced parts of usual care. Some studies (7/24, 29%) involved active research team engagement by checking in with their participants to, among others, troubleshoot (technical) issues [24,25,30,37,38,40,43,45]. Preuse instructions were provided in some of the studies (9/24, 38%) [24,25,28,32,35,36,41,44,45,48]. Control conditions were typically usual care. Three studies chose an active control group in which participants received a nurse-led education program or discussion with nurses [28], a self-help book [47], or intensive specific health guidance [37]. One study [35] assigned their participants to the control or intervention condition based on CA use. Only 8% (2/24) of the studies [26,48] reported the technological experience of their participants.

**Table 3:** Study design and sample characteristics.

Study, year, and country		Study design, follow-up, and study objective	Sample characteristics (age in y, mean [SD], gender, level of education, and technology skills)	Description of the study condition
**Atrial fibrillation**				
	Guhl et al [41], 2020; United States	Randomized controlled trial,^a^ 30 d, to measure acceptability and adherence and to assess its effectiveness to improve health-related QoL^b^ and adherence.	Age: 72 (9); gender: 52% women; level of education: 28% high school and vocational, 11% some college, 28% bachelor’s degree, 33% graduate; technology skills: NR^c^	Reimbursement: NR, IG^d^ (N=61): use of the app and Kardia device. Preuse instruction: trained until achieved proficiency in using the smartphone, relational agent, and Kardia device, including an orientation to the phone and app. CG^e^ (N=59): Usual care. Preuse instruction: recording an electrocardiogram with Kardia under study personnel supervision.
**Cancer**				
	Tawfik et al [28], 2023; Egypt	3-arm randomized controlled trial, 4 mo, to examine the effects compared to nurse-led education on the effectiveness of self-care behaviors and the frequency, severity, and distress of chemotherapy side effects.	Breast cancer. Age: IG 44 (6), CG nurse-led 46 (9), and CG usual care 45 (8); gender: 100% women; level of education: 40% read and write, 16% basic education, 30% secondary diploma, 14% higher education; technology skills: NR	Reimbursement: NR. IG (N=50): received empowerment-based educational program via ChemoFreeBot. Preuse instruction: taught about the chatbot, its objectives, and that they would be chatting to an automated system. CG, nurse-led (N=50): 3 face-to-face group teaching sessions (45 min each) and a brochure. CG, usual care (N=50): discussing general knowledge of self-care behaviors regarding managing chemotherapy side effects with a nurse, varies in depth across individuals.
	Greer et al [29], 2019; United States	Randomized controlled cross-over trial^a^, 4 wk, to evaluate the engagement and usability of Vivibot and its preliminary effects of positive psychology skills delivered on psychosocial well-being outcomes in young adults treated for cancer.	Age: 25 (3); gender: 46% women; level of education: 0% >high school, 12% high school graduate and general educational development, 32% some college, 8% 2-y college degree, 40% 4-y college degree, 8% master’s degree; technology skills: NR	Reimbursement: US $20 Amazon gift card for each completed online survey. IG (N=25): access to the Vivibot. CG (N=26): waitlist, access to Vivibot after 4 wk.
	Schmitz et al [25], 2023; United States and Caru et al [24], 2023; United States	Partial cross-over randomized trial, 6 mo, Schmitz: To test the feasibility, acceptability, and initial efficacy of a supportive care intervention called nurse AMIE^f^. Caru: To present step counts data as exploratory evidence to document the impact of virtual-assistant technology on enhancing daily step count in women with metastatic breast cancer.	Metastatic breast cancer. Age: all 53 (11), IG 55 (10), CG 52 (8); gender: 100% women; level of education (all): high school 17%; some college 26%; 4-y degree or more 57%, level of education in IG: high school 10%; some college 33%; 4-y degree or more 57%, level of education in CG: high school 24%, some college 19%, 4-y degree or more 57%; technology skills: NR	Reimbursement: NR, IG (n=21): access to nurse AMIE. Check-in: weekly calls during the first 3 mo, which covered general queries regarding well-being and symptom checks and troubleshooting technology issues. Preuse instruction: participants engaged in an orientation session. CG (n=21): waitlist, first 0-3 mo usual care. After 3 mo access to intervention.
	Gomaa et al [30], 2023; United States	Single-arm study^a^, 2 mo, to evaluate its practical implementation, user acceptance, and its potential to enhance QoL, patient activation, and symptom distress management.	Malignant gastrointestinal cancer. Age: 61 (12); gender: 56% women; level of education: high school 18%, college graduate 47%, postgraduate school 32%; unknown 3%, technology skills: NR	Reimbursement: gift cards valued at US $20, US $30, and US $40 for successive time points. IG (n=34): Received pro- and interactive text messages 3 times a week, a chatbot interface to monitor participant symptoms, and immediate self-care feedback. Check-ins: biweekly in-person meetings or telephone calls to ensure participant progress and address any queries. In addition to usual care.
	Huang et al [31], 2023; Taiwan	Retrospective cohort study, 1 y (follow-up per participant varies depending on chemotherapy starting date). To evaluate whether a chatbot-based collection of patient-reported symptoms during chemotherapy treatment, with automated alerts to clinicians for severe or worsening symptoms, decreases emergency department use and reduces unscheduled hospitalizations	Gynecologic cancer. Age: IG 48 (9) and CG 64 (11); gender: 100% women; level of education: NR; technology skills: NR	Reimbursement: NR, IG (N=20): access to the chatbot program. CG (N=43): usual care consisting of standard care procedures at the hospital, including discussing symptoms and documenting them in the medical record during clinical encounters between patients and their oncologists. Patients with direct concerns about symptoms were also encouraged to initiate telephone contact with cancer managers between visits.
	Albino de Queiroz et al [40], 2023; Brazil	Prospective nonrandomized clinical study, 8 wk, to evaluate the benefits of the SMT^g^ model to patients regarding adverse effects, treatment, and QoL.	Colorectal cancer. Age: IG 49.7 (13.4) and CG 50.8 (13.5). Gender: IG 61.5% female and CG 29.4% female. Level of education: NR. Technology skills: NR	Reimbursement: NR, IG (N=13); monitoring proposed in this study. Check-ins at wk 2 and 6 addressed user doubts and provided guidance on app and device use. Participants with low engagement received alerts encouraging them to adhere to the intervention. CG (N=17): traditional monitoring during the active cancer treatment phase.
**Chronic illness**				
	Fang et al [42], 2018; Australia	Randomized controlled trial^a^, 3 wk, to describe the solution of using an avatar-based reminder app and the results of an initial trial.	People taking supplements. Age (range): 18-70, gender: NR, level of education: NR, technology skills: NR	Reimbursement: NR, IG (N=11); access to a simple version of the app with limited interaction via a study iPad. Participants filled weekly Zip-lock bags with their supplements. CG (N=13): an electronic pillbox with alarm functions and compartments for participants to fill with their supplements weekly.
**Chronic kidney disease**				
	Chen et al [32], 2023; Taiwan	Pre-post intervention design^a^, 3 mo, to gather the data required to develop an intervention program of CIM-SHE^h^ for patients with chronic kidney disease.	Age: 55 (10); gender: 36% women; level of education: 36%≤high school; 64%≥college; technology skills: NR	Reimbursement: gift certificate of NT $300 (approximately US $10) after completing the pretest, intervention, and posttest. IG (n=60): access to the app. Chatbot manager actively interacts with participants during the intervention to improve their learning motivation. Preuse instruction: face-to-face meeting with the research team for advice on operating the chatbot and interacting with the instructor and other participants.
**Chronic pain**				
	Meheli et al [27], 2022; United States	Retrospective observational study, 1 y, to evaluate the perceived needs, engagements, and effectiveness of the mental health app Wysa regarding mental health outcomes among real-world users who reported chronic pain and engaged with the app for support.	Age: NR; gender: NR; level of education: NR; technology skills: NR	IG: Textual snippets (n=2194) of Wysa app users that reported chronic pain. CG: textual snippets of Wysa app users without chronic pain (only used for testing the app engagement or disengagement).
	Cheng et al [26], 2023; United States	Single-site, single-arm, prospective cohort study^a^, 1 mo, to identify behavioral mechanisms that may mediate changes in mental and physical health associated with the use of Wysa for chronic pain during orthopedic management of chronic musculoskeletal pain.	Age: 59 (14); gender: 70% women; level of education: NR. Technology skills with technological experience measured as self-reported general smartphone use patterns: 57% independent downloading and using apps; 20% need help with downloading and using apps; 17% smartphone users but not for apps; 7% never use a smartphone.	Reimbursement: US $40 gift card. IG (N=30): Wysa for Chronic Pain digital intervention in addition to usual orthopedic care, which includes analgesic medication, physical therapy, and interventional spine procedures, as appropriate.
	Hauser-Ulrich et al [33], 2020; Switzerland	Randomized controlled trial^a^, 8 wk, to describe the design and implementation of Selma and present findings from a trial that evaluated effectiveness, acceptance, and adherence.	Age: 44 (13); gender: 80% women; level of education: 12% obligatory and high school, 32% matriculation and A-level, 21% higher vocational training, 35% university. Technology skills: NR	Reimbursement: NR, IG (N=59): 8-wk digital coaching program with a fully automated CA mediating coping strategies and psychoeducation to support pain self-management. CG (N=43): wait-list, completed the introduction process with the chatbot at day 0 and received weekly motivational messages from the chatbot. Were able to access the app after the waiting time (8 wk).
Diabetes				
	Krishnakuma et al [34], 2021; South Asia	Pre-post study, 16 wk, to investigate the real-world effectiveness of the Wealthy CARE digital therapeutic for improving glycemic control among the South Asian population of Indian origin.	Type 2 diabetes mellitus. Age: 41 (CI 49-53); gender: 31% women; level of education: NR; technology skills: NR	IG (N=102): 16-wk structured lifestyle coaching through the Wellthy CARE digital therapeutic app.
	Roca et al [44], 2021; Spain	Pre-post study,^a^ 9 mo, to validate the effectiveness of a health care virtual assistant, integrated within messaging platforms, with the aim of improving medication adherence in patients with comorbid type 2 diabetes mellitus and depressive disorder.	Age: 64 (9); gender: 69% women; level of education: NR, technology skills: NR	IG (N=13 patients, N=5 health care professionals): access to the app via signal. Preuse instruction: during a medical appointment, nurses assisted with app download, explained the initial interaction with the virtual assistant, registered the patient, and configured the medication and reminder functions.
	Gong et al [43], 2020; Australia	Randomized controlled trial, 12 mo, to evaluate the adoption, use, and effectiveness of the My Diabetes Coach program designed to support diabetes type 2 self-management in the home setting.	Age: 57 (10); gender: 42% women. Level of education: 19% secondary high school or lower, 31% technical apprenticeship or diploma, 21% bachelor’s degree; 19% postgraduate degree or higher; technology skills: NR	IG (N=93): My diabetes coach program and (optional) blood glucose meter with Bluetooth. Encouraged to regularly access the user guide and website and to join the discussion forums. Check-in: phone call encompassing a brief, structured interaction with the program coordinator (at 1, 4, 8, 12, and 24 wk) for technical assistance, to answer questions, and to encourage program use. CG (N=94): encouraged to continue routine diabetes self-care (including access to health care services and resources via NDSS^i^ and diabetes not-for-profit organizations). Received a quarterly project newsletter to maintain their interest in the study. Program access after the study duration, if desired.
	Bruijnes et al [47], 2023; unclear	Double-blinded between-subject study,^a^ 3 wk, to determine the feasibility and preliminary efficacy of an automated CA to deliver to people with diabetes personalized psychoeducation on dealing with (psycho-) social distress related to their chronic illness.	Age: IG 37 (15), CG 40 (16), all 39 (16); gender: IG 57% women, CG 37% women, all 47% women; level of education: NR; technology skills: NR	Reimbursement: a minimum of £6 (approximately US $7.50) per hour, with increasing bonuses for completing consecutive sessions to reduce attrition. IG (N=79): 3 sessions in which the CA iterates over providing advice, evaluating the usefulness of the previous advice, and giving alternative advice, or working on another issue. Each session was separated by at least 1 wk. CG (N=77): 3 sessions in which they read or reread a self-help text from the book *Diabetes Burnout* to deal with interpersonal distress and friends and family distress (similar content but not personalized). Each session was separated by at least 1 wk.
	Nassar et al [35], 2023; United States	Study design: NR, 9-12 mo, to provide support for T2DM^j^ management, to improve self-care confidence, and to explore impact on A_1C_.	Age: 58 (10.6); gender: 60% women; level of education: NR; technology skills: NR	IG (N=58): patients that completed more than 1 chat. Preuse instruction: participants were informed they were not talking to a live human and that the chatbot was not monitored 24 h a day. CG (N=36): A priori enrollee who completed none or only a single chat, as the first chat consisted of administrative information alone and did not deliver any education content.
HIV				
	Dworkin et al [45], 2019; United States	Pre-post study,^a^ 3 mo, to explore the acceptability, feasibility, and preliminary efficacy of the effect of My Personal Health Guide on adherence.	Young men positive with HIV that have sex with men. Age (range) 29 (18-34); gender: 0% women; level of education: 33% less than a high school diploma, 67% college or more; technology skills: NR	Reimbursement: baseline and follow-up visit US $50; returning to study site to resolve issues in case of app deletion or phone loss, US $15. IG (N=43): access to health coach app. Preuse instruction: demonstration of app functions, reminder settings, encourage app use, and answering questions. Check-in: phone call by project staff (after mo 1 and 2) to troubleshoot technical problems.
**Hypertension**				
	Echeazarra et al [36], 2021; Spain	Randomized controlled trial, 2 y, to evaluate the feasibility of developing the chatbot, assess its effectiveness on blood pressure checking and knowledge improvements on best-practice or self-management blood-pressure measurement procedures and investigate advantages of using the CA.	Age: 52.1; gender: 42% women; level of education: 25% basic studies, 22% medium studies, 31% vocational training, 13% university graduated, 9% not specified. Technology skills: NR	IG (N=55): Access to Tensiobot. Preuse instruction: the nurse helps to download and install the Telegram app and with registering to and use of Tensiobot. CG (N=57): receives a written procedure on how to self-monitor blood pressure.
	Sakane et al [37], 2023; Japan	Randomized controlled trial,^a^ 12 wk, to determine the efficacy of the KENPO app in facilitating weight loss in Japanese adults with obesity and hypertension.	Age: 52 (7); gender: 45% women; level of education: NR; technology skills: NR	Initial counseling by a registered and trained dietician consisting of a briefing about the patients’ health condition and lifestyle and instructions to set achievable personalized behavioral goals. Receive self-monitoring devices (Bluetooth weighing scale, pedometer, and upper arm blood pressure monitor). Check-in: e-mail support at 2, 6, and 12 wk. Kind of support not specified. IG (N=39): access to the app. CG (N=39): active control, usual support based on intensive specific health guidance. Recommended to record step count and body weight daily and measure blood pressure in the morning and evening.
**IBS^k^**				
	Hunt et al [38], 2021; United States	Randomized controlled crossover trial, 3 mo, to evaluate the efficacy of Zemedy to apply cognitive behavioral therapy to IBS.	Age: 32 (10); gender: 75% women; level of education: NR; technology skills: NR	Reimbursements: US $20 in Amazon credit upon completion of each round of questionnaires. IG (N=62): Access to app, entire intervention delivered within the app, no human involvement. In case of technical difficulties, users could reach out to technical support. Check-in: via email at 4 wk from a research coordinator providing general encouragement to continue working through the app. CG (N=59): waitlist, access to app after 8 wk. Check-in: received a single email at 4 wk to hang in there.
**Kidney failure**				
	Cheng et al [39], 2023; Taiwan	Study design: NR, 12 mo, to improve the peritonitis incidence by assisting PD^l^ treated patients with knowledge and quality of self-care by using AI^m^ combined with social media	Age (percentage of participants within age range): 21-50 (42%), 51-70 (47%), >70 (11%); gender: 53% women. Level of education: elementary school (8%), junior high school (7%), senior high school (28%), university (44%), graduate school (13%); technology skills: NR	IG (use data N=440, questionnaire data N=297): introduced to the PD AI-Chatbot
**Overactive bladder**				
	Sheyn et al [48], 2024; United States	Prospective observational study,^a^ 8 wk, to evaluate the efficacy of a digital CA (CeCe) for the treatment of overactive bladder.	Age (median): 61 (IQR 52-67); gender: NR; level of education: NR; technological experience, median: MDPQ-16^n^: 34 (IQR 25-37), CPQ^o^: 26 (IQR 19-30)	Reimbursement: US $175 was compensated throughout the study to ensure adherence: US $50 at completion of the voiding diary and initial set of questionnaires (d 1-3), US $50 at completion of the 4-wk voiding diary and questionnaires, and US $75 at completion of the 8-wk voiding diary and questionnaires. IG (N=29): CeCe treatment program. Preuse instruction: the research coordinator assisted with setting CeCe up on the patient’s mobile phones, enrolled them in CeCe, and showed how to use the app.
**Primary headaches**				
	Ulrich et al [46], 2024; Switzerland, Germany, and Austria	Unblinded randomized controlled trial, 24 to 60 d (intervention group); 60 d (control group). To develop a smartphone-based and CA-delivered intervention for people with headache and to evaluate the smartphone-based and CA-delivered interventions’ effectiveness, engagement, and acceptance.	Age: 39 (12); gender: 91% women; level of education: no education 3%, obligatory or high school 1%, vocational training and high school 30%, higher vocational training 15%, university (of applied sciences) 52%, technology skills: NR	IG (N=110): unguided use of the intervention. CG (N=88): waitlist, unguided use of the intervention after 42 d. Received a weekly reminder from the CA during the 42-d waiting period (not described what these reminders include).

^a^Reported as pilot study.

^b^QoL: quality of life.

^c^NR: not reported.

^d^IG: intervention group.

^e^CG: control group.

^f^AMIE: Addressing Metastatic Individuals Everyday.

^g^SMT: Smart Monitoring Tool.

^h^CIM-SHE: Chat-based Instant Messaging Support Health Education.

^i^NDSS: National Diabetes Service Scheme.

^j^T2DM: type 2 diabetes mellitus.

^k^IBS: inflammatory bowel disease.

^l^PD: peritoneal dialysis.

^m^AI: artificial intelligence.

^n^MDPQ-16: Mobile Device Proficiency Questionnaire.

^o^CPQ: Computer Proficiency Questionnaire.

**Table 4:** Outcome measures.

Study		Outcome measures				Primary outcomes
		Efficacy or effectiveness	Feasibility	Acceptability	X>Y: X significantly improved compared to Y. X=Y: No significant difference.	
**Atrial fibrillation**						
	Guhl et al [41]	Health-related QoL^a^: Atrial fibrillation effect on QoL measure, pre and post, self-reported at clinical site.^b^ Medication adherence: 2 questions (forget or not take medication), pre and post, self-reported at clinical site.	Participant flow diagram (enrolment, exclusion, attrition, and retention rate). Intervention adherence: median number of conversations and days of use, and mean total interaction duration, duration per conversation, and number of completed modules, and number of reported symptoms, not described as outcome in the methods but reported in the results.	Acceptability: survey including free-text responses (eg, “overall impressions of Tanya”) and closed questions (usefulness, informativeness, trustworthiness, easiness, and repetitiveness of the CA^c^), post, self-reported at clinical site.	Health-related QoL: IG^d^>CG^e^	
**Cancer**						
	Tawfik et al [28]	Frequency, severity, and distress of physical and psychological chemotherapy-related side effects: Adapted version of the MSAS^g^, pre and post, self-reported, collection NR^b,f^. Effectiveness of self-care behavior: Modified SCBD^h^, pre and post, self-reported, collection NR.^b^	N/A^i^	Usability: CUQ^j^, post, self-reported, collection NR.	Frequency, severity, and distress of physical and psychological chemotherapy-related side effects: IG>CG. Effectiveness of self-care behavior: IG>CG	
	Greer et al [29]	Pre and post, self-reported in app: Anxiety: 4-item PROMIS^k^ Emotional Distress-Anxiety Depression: 4-item PROMIS Emotional Distress-depression Positive and negative affect: modified Differential Emotions Scale Emotion: ratings, daily, self-reported in app.	Participant recruitment and flow (enrolment, attrition, exclusion, and retention rate). Engagement (user data): Conversation history per session (=≥2 user inputs in 5 min) Number of sessions Total interaction time Number of engaged sessions (=user completes daily emotion rating).	CA feedback: Ratings of helpfulness of each lesson, and survey assessing if users would recommend to a friend and why after lesson 7, self-reported in app.	N/A	
	Schmitz et al [25]; Caru et al [24]	Schmitz et al [25]: Assessed at baseline, 3 mo follow-up and post intervention, online via, video conference: Physical function: 5-times sit-to-stand chair test. Quality of life: SF-36^l^. Pain: Brief Pain Inventory and pain-subscale of the SF-36. Sleep: Sleep disturbance scale. Fatigue: vitality subscale of the SF-36. Distress: distress thermometer. Caru et al [24]: Step count: pedometer measuring from waking up until going to bed, daily, self-reported in app.	CONSORT^m^ diagram (enrolment, attrition, exclusion, and retention rate). Feasibility: Number of days out of the first 90 d of exposure to Nurse AMIE^n^ that patients logged in.	Helpfulness of the intervention: one question, daily, in app. Usability: the CSQ^o^, the Credibility and Expectancy Questionnaire, and the user version of the mobile app rating scale. At 3 mo, online video conferencing. Acceptability: % of the eligible patients approached who agreed to participate.	N/A	
	Gomaa et al [30]	Pre and post, online via web-based platform or on paper during clinical visits: QoL: Functional Assessment of Cancer Therapy Symptom experience and burden of patients: MSAS Perceived capacity of patients in managing own health: Patient activation measure. Perceived social support: Multidimensional Scale of Perceived Social Support. Preparation for chemotherapy and radiotherapy: Cancer Treatment Survey instrument.	Feasibility: study accrual and attrition rates. Use with use data: Engaged to text back ≥1 time. Interaction range (number of messages). Most frequently used keyword. Most frequently reported symptoms.	Acceptability: intervention satisfaction with self-made 5-item ratings on a 1-5-point scale evaluating usability and acceptability, post, online via web-based platform or on paper during clinical visits. Acceptability: semistructured interviews on user experience and feedback, post, by phone.	N/A	
	Huang et al [31]	ED^p^ visits: adjusted incidence rate ratio of ED visits after initiation of chemotherapy, method NR.^b^ Unscheduled hospitalizations: adjusted incidence rate ratio of unscheduled hospitalizations after initiation of chemotherapy, method NR.	Number of consultations with the intervention, use data. Percentage of consultations that require further in-person communication, use data.	Patient satisfaction (method NR, not described as outcome in the methods but reported in the results).	ED visits: IG>CG	
	Albino de Queiroz et al [40]	QoL: QLQ-C30^q^ and QLQ-CR29^r^, at 0, 4, and 8 wk, self-reported within the CA, IG only. Eating habits and physical activity: “Food Guide: how to have a healthy diet questionnaire,” pre and post intervention, self-reported within the CA, IG only. Signs, symptoms, and adverse effects: Data from the CG were extracted from the patient's medical records, and from the IG were obtained from the patient's self-report, not described as outcome in the methods but reported in the results.	Engagement: Time of use of app and wearable device. Quality of the user-reported data. Patient interactions with the model: Number of interactions performed Number of users interacting with each item. Number of generated notifications to the care team. Accrual and attrition rate (not labeled as such by the authors).	Usability: SUS^s^, post, self-reported within the CA. User experience: User Experience Questionnaire, post, self-reported within the CA.	N/A	
**Chronic illness**						
	Fang et al [42]	Medication adherence: adherence rate using weekly pill counts, pre and post, self-reported, collection NR.	Study recruitment	User experience: ratings (post) and interviews (pre and post), face-to-face.	N/A	
**Chronic kidney disease**						
	Chen et al [32]	Communicative literacy: survey based on the chronic kidney disease knowledge scale with 2 items added evaluating disease-specific health literacy and disease knowledge, pre and post, by face-to-face or telephonic interviews, depending on the participants’ willingness.	Study enrolment (enrolment, attrition, exclusion, and retention rate)	Acceptability: SUS evaluating usability, weeks 1, 4, and 12 and post, by face-to-face or through telephonic interviews, depending on the participants’ willingness.	N/A	
**Chronic pain**						
	Meheli et al [27]	Depression (n=69): PHQ-9^t^, pre and post, self-reported in app. Anxiety (n=57): GAD-7^u^, pre and post, self-reported in app. Collection moment varies per individual, based on first and last assessments within the 1-y study.	App engagement or disengagement: with use data. Use frequency. Intensity of user engagement: Number of initiated and completed interactions per user. Points of disengagement: instances where users stop communicating or engaging further with the CA.	Perceived needs of users with chronic pain: textual snippets from conversations.	N/A	
	Cheng et al [26]	Changes in self-reported mental and physical health with the adult PROMIS computer adaptive test Anxiety (version 1.0), Depression (version 1.0), Pain Interference (version 1.1), and Physical Function (version 2.0). Pre and post, post self-reported online and pre-intervention withdrawn from electronic medical record.	Inclusion flow sheet (enrolment, attrition, exclusion, and retention rate). Use of the intervention with CA interactions (digital and human coach): time-stamped participant use data.	NR	N/A	
	Hauser-Ulrich et al [33]	Pre and post, self-reported in app: Pain-related impairment: 7-item subscale of the Brief Pain Inventory.^b^ Pain intensity: German Pain Questionnaire. General well-being: Marburger Screening for Habitual Well-being. Intention to change behavior: health action process	Participant flowchart (enrolment, attrition, exclusion, and retention rate). Intervention adherence: Use data, the ratio of conversations replied to by participants and all conversations initiated by Selma.	Acceptability: survey assessing usefulness, ease of use and enjoyment, satisfaction with intervention duration and number of messages, sufficiency of the content, net promotor score, and what users like most and what they want to see improved, post, self-reported in app. Working alliance: context-adapted German version of the Working Alliance Inventory Short Revised, post, self-reported in app.	Pain-related impairment: IG=CG	
**Diabetes**						
	Krishnakuma et al [34]	HbA_1c_^v^: Independent pathological laboratory test, pre and post.^b^ Pre and post, self-monitoring data in app: Fasting blood glucose Postprandial blood glucose BMIw Weight	Participant recruitment and retention flowchart (enrolment, attrition, exclusion, and retention rate). Use: average time spent with the chatbot and health coach with use data. Program engagement: number of interactions with the health coach and AI-powered chatbot with use data.	NR	HbA_1c_: post > pre	
	Roca et al [44]	HbA_1c_: Laboratory blood test, at pre and post. Pre and post, telephone interview: Depression: PHQ-9 Medication adherence: Medication Possession Ratio, threshold 80%. Health care resources impact: Number of medical appointments per mo. Experienced changes in medication adherence by patients with guided telephone interviews (closed questions) at post. Changes in patient medication adherence by health care professionals: self-assessment at post.	Flow diagram of patient selection and completion of the pilot study (enrolment, attrition, exclusion, and retention rate)	Patient user experience: Guided interviews in primary health care centers, 3 mo to evaluate general impression (ease of learning, usefulness, use frequency, covered needs in medication reminders, and easiness of functionalities), observed problems (use, reminders, and vocabulary), and other suggestions (unmet needs and opinions). Health care provider user experience: Self-administered questionnaire assessing ease of use, usefulness, monitoring features, willingness to continue, patient motivation and dissatisfaction, at post. Patients’ use and acceptance of the virtual assistant: Use data to evaluate CA use (daily interactions, functionalities, and answered reminders), interaction preference (numeric and text), acceptance (uninstalls), and usefulness (misunderstandings)	N/A	
	Gong et al [43]	HbA_1c_: Pathology blood test, requested from general practitioner, pre and post, in clinic.^b^ Pre and post, self-report survey, online: Health-related QoL: Assessment QoL-8D scaleb Anxiety and depressive symptoms: Hospital Anxiety and Depression Scale. Diabetes-specific distress: Problem Areas in Diabetes scale Body weight assessed by general practitioner, pre and post, in clinic.	Enrollment, randomization, and follow-up of study participants. (attrition, exclusion, and retention rates). Adoption and use: use data to evaluate duration of chats and number of blood glucose uploads, technical alerts, clinical alerts, and completed chats	NR	HbA_1c_: IG=CG. health-related QoL: IG>CG	
	Bruijnes et al [47]	Social diabetes distress: a survey generated from subscales of the type 1 Diabetes Distress Scale and type 2 Diabetes Distress Scale adjusted to 3-wk intervention, post, self-reported online via email invitation. Feeling of being heard: FBH^w^ questionnaire on 7-point Likert scale, post, self-reported online via email invitation.	Exclusion and attrition rate	Attitude toward the intervention: CSQ 8 items, post, self-reported online via email invitation. Usefulness of the implementation of the CA: SUS, post, self-reported online via email invitation.	N/A	
	Nassar et al [35]	Glycemic outcomes (A_1C_): extracted from the electronic medical record at pre (most recent A_1C_ measure up to 7 d after enrolment) and post (first A_1C_ measure occurring at least 6 wk after the enrolment date).^b^ Self-care confidence: one question “I feel confident that I can control and manage most of my health problems,” every 3 mo, self-reported in app.	Enrollment and unenrollment (ie, opting to unenroll through the chatbot or by asking the staff). Engagement: the proportion of eligible users who complete an engagement behavior, based on use data from the CA dashboard. Activation: use data evaluating the enrollment ratio (users who clicked through terms and conditions vs those who agreed to enroll), simple engagement (users who returned within 1 mo of their first chat), active engagement (users who completed more than half of their invited chats). Number of completed chats: use data. Red flags: use data evaluating the number of red flags, users with red flags, and reasons for red flags.	Satisfaction: questions (eg, helpfulness of today’s chat) and overall satisfaction with the length and frequency of the chat and quarterly survey, self-reported in app. User experience: Chat comments and posts.	A_1C_: IG>CG	
**HIV^y^**						
	Dworkin et al [45]	Medication adherence: the percentage of users with pill count–based adherence ratio >80%, pre and post, collection method not specified NR.^b^. Pre and post, self-reported, at clinical site: Medication adherence: Missed doses in past 4 wk. Self-efficacy: 2 questions derived from the Coping Self-Efficacy scale and 1 overarching question (confidence in taking medication consistently). HIV information evaluating knowledge of information taught in the app, method NR.	Enrollment, attrition, exclusion, and retention rate. Methods NR: Responsiveness to study check-in calls Technical difficulties Phone losses App deletions before follow-up Users that completed the surveys and pill count.	User experience: Likert-scale survey (use easiness, willingness to continue using after study, and perceived value of the functions, feelings of discomfort, being embarrassed, and in control of their health and being cared for by the avatar, and whether they recommend the app to an HIV-infected friend), post, in-clinic. Extent of app use: Use data.	Medication adherence: post=pre.	
**Hypertension**						
	Echeazarra et al [36]	Monitoring adherence and quality: contrast between Holter device measurement assisted by nurse and self-monitored blood pressure, post. Blood pressure self-monitoring knowledge and skill: checklist assessed by nurse during clinical appointment, pre and post. Patient adherence: number of correct blood pressure checks (method unspecified)	NR	User satisfaction: satisfaction survey, including questions about ease of use, usefulness, preference, and stopped using the app, post, and method NR	N/A	
	Sakane et al [37]	Weight loss and BMI: weighing scale, daily, self-monitored and uploaded from device to the cloud. Daily, self-monitored and uploaded from device to the cloud: Blood pressure: Upper arm blood pressure monitor. Daily steps: Pedometer Healthy habits: Web-based self-administered 10-item survey, pre and post.	CONSORT flow diagram (enrolment, attrition, exclusion, and retention rate). Device adherence: number of uploads of weight, blood pressure, and steps, self-monitoring data, weekly.	NR	N/A	
**IBS^x^**						
	Hunt et al [38]	Pre and post, online, self-reported survey: QoL: IBS-QoL.^b^ Symptom severity: Gastrointestinal symptom rating scale-IBS.^b^ Fear, avoidance of food and life interference, and loss of pleasure form eating: Fear of food questionnaire. Gastrointestinal -specific anxiety: visceral sensitivity index Gastrointestinal specific catastrophic cognitions: Gastrointestinal cognitions questionnaire Depression, anxiety, and stress; Depression Anxiety Stress Scale. Depression: PHQ-9	CONSORT diagram of participant flow (enrolment, attrition, exclusion, and retention rate). Received dose: number of modules completed with use data.	NR	QoL: IG>CG after intervention of 8 wk. Symptom severity: IG>CG, after intervention of 8 wk.	
**Kidney failure**						
	Cheng [39]	Infection rate: Differences in PD^y^-related infections, during app use, method NR.	User clicks: Number of clicks by patients by use data.	Acceptance and satisfaction OR user satisfaction and willingness to use the PD AI^bb^ Chatbot during the COVID-19 pandemic OR Patient satisfaction: Likert-scale based satisfaction self-report survey at 3 mo.^b^, qualitative evaluation (NR)	Satisfaction: overall satisfaction=4.5 out of 5. High satisfaction (<4 points) was reported for (1) 93.6% of users who believed that the PD AI chatbot could help reduce the length of their hospital stay during OPD^aa^ visits. (2) 98.6% of users reported receiving health education content immediately; (3) 94.9% of users found the chatbot easy to use; and (4) 98.6% of users expressed their desire to continue using the chatbot.	
**Overactive bladder**						
	Sheyn et al [48]	QoL: Change in the International Consultation on Incontinence-Overactive Bladder QoL Questionnaire, pre and post, self-reported in app.^b^, self-reported in app*:* Health status: SF-36, pre and post. Anxiety: GAD-7, pre and post. Overactive-bladder symptoms: Voiding diaries at weeks 1, 4, and 8. Patients believe in the treatment efficacy: Patient Global Impression of improvement, post.	Flowchart of participant selection (enrolment, attrition, exclusion, and retention rate)	Usability: SUS, post, self-reported in app.	QoL: post>pre.	
**Primary headaches**						
	Ulrich et al [46]	Mental well-being: composite score of the PHQ-9 and GAD-7, pre and post, self-reported in app.^b^ Pre and post, self-reported in app: Depression: PHQ-9. Anxiety: GAD-7 Somatic symptoms: PHQ-15. Stress: Perceived stress scale-10, German version. Self-efficacy: German short form of the Headache Management Self-efficacy scale. Absenteeism and presenteeism: Questions form the Migraine Disability Assessment. Adverse events and impression of change: Patient Global Impression of Change scale, modules 1 and 7, self-reported in app. Participants’ intention to change behavior: Survey, self-reported in app at baseline. Pain coping: Fragebogen zur Erfassung der Schmerzverarbeitung first part, pre/post, in self-reported in app. Application of BCTs: health action process approach stage of change-based survey, self-reported in app at onboarding, modules 3 and 6.	Participant flowchart (enrolment, attrition, exclusion, and retention rate). Extent of use: use data assessing the time spent on in-app relaxation and imagination exercises, the number of inactivity reminders, and days taken to complete a coaching module. Engagement with use data: percentage of answered conversational turns between the participant and the CA (higher=higher engagement). Intended use with use data: participants that completed the outro.	Subjective experience, self-reported in app: Engagement and acceptability: 4-items of the German Group Therapy Session Evaluation by Patients, after completing a session and a unit. Perceived enjoyment: “Did you enjoy the last unit?” Acceptance: open questions “What did you like most about the BalanceUP app?” and “What would you like to see improved about the BalanceUP app?” post, self-reported in app. Engagement and acceptance, self-report in app: Feasibility and acceptance—engagement, information, perceived quality, and perceived impact: modified and translated version of the Mobile App Rating Scale, post. Working alliance: Session Alliance Inventory short-revised patient version (in German) and 3 item bond and 3 item task and goal scale at the end of modules 2, 4, and 6. Commitment to behavior change: “How committed are you towards changing your behavior?” at onboarding and in modules 3 and 6. Intention to change behavior: health action process approach model-based survey at onboarding, modules 3 and 6.	Mental well-being: IG>CG	

^a^QoL: quality of life.

^b^Primary and coprimary outcomes.

^c^CA: conversational agent.

^d^IG: intervention group.

^e^CG: control group.

^f^NR: not reported.

^g^MSAS: Memorial Symptoms Assessment Scale.

^h^SCBD: self-care behaviors diary.

^i^N/A: not applicable.

^j^CUQ: Chatbot Usability Questionnaire.

^k^PROMIS: Patient-Reported Outcomes Measurement Information System.

^l^SF-36: 36-Item Short Form Survey.

^m^CONSORT: Consolidated Standards of Reporting Trials.

^n^AMIE: Addressing Metastatic Individuals Everyday.

^o^CSQ: Client Satisfaction Questionnaire.

^p^ED: emergency department.

^q^QLQ-C30: Quality of life of Cancer Patients survey.

^r^QLQ-CR29: Quality of Life of the Colorectal Cancer patients survey.

^s^SUS: System Usability Scale.

^t^PHQ-9: Patient Health Questionnaire-9.

^u^GAD-7: General Anxiety Disorder-7.

^v^HbA_1c_: hemoglobin A_1c_.

^w^FBH: Feeling of Being Heard.

^x^IBS: inflammatory bowel disease.

^y^PD: peritoneal dialysis.

^z^AI: artificial intelligence.

^aa^OPD: outpatient department.

#### Efficacy or Effectiveness Outcomes

Clinical manifestations of the disease were assessed most often (11/24, 46%). These include physical function examined with a chair test in cancer and a survey in chronic pain [25,26]; symptoms of cancer, pain, headaches, overactive bladder, and irritable bowel syndrome evaluated with surveys or a diary [25,26,30,33,38,46,48]; blood glucose levels determined with blood tests in diabetes [34,35,43,44]; BMI or weight assessed by a general practitioner in clinic or self-monitored data using a weighing scale in diabetes type 2, obesity, and hypertension [34,37,43]; and blood pressure examined using a blood pressure monitor in obesity and hypertension [37]. In addition, medical complications were assessed using unspecified methods, such as infection rate from peritoneal dialysis for chronic kidney disease [39] and department emergency visits and unscheduled hospitalizations of patients with cancer [31]. Side effects of chemotherapy for cancer were measured once with a survey [28].

More general outcomes were measured with a variety of (disease-specific) surveys. General outcomes encompassed the (health-related) QoL of patients with colorectal cancer, irritable bowel syndrome, atrial fibrillation, diabetes, and an overactive bladder [38,40,41,43,48] and mental health–related outcomes, with anxiety or depression being the most frequent, in patients with cancer, chronic pain, irritable bowel syndrome, diabetes, headaches, or an overactive bladder [25-27,29,38,43,44,46-48].

Self-management [30] or related concepts such as self-care confidence [35] and self-efficacy [45,46] were assessed using standardized questionnaires [30,45,46] or by adding one question to the test battery [35]. In addition, 13% (3/24) of the studies measured the effect of education with surveys assessing communicative literacy [32], health literacy [45], and blood pressure self-monitoring knowledge and skills [36]. Moreover, 8.3% (2/24) of the studies evaluated the participants’ intention to change their behavior [33,46]. Some (8/24, 33%) studies assessed changes in behavior with step counts using a pedometer [24,37], healthy habits using a survey [37], changes in food consumption and physical activity using a survey [40], application of BCTs using a survey [46], adherence to medication using pill counts [42,45], questions about (changes in) adherence [41,44], adherence to monitoring using number of correct measures [36], and monitoring quality by comparing self-assessed measurement to golden standards [41,42,44,45]. The effectiveness of self-care behaviors was assessed once [28]. The impact of the intervention on health care use was measured twice [31,44].

#### Feasibility Outcomes

One study [30] reported accrual and attrition rates as feasibility measures. Another study [25] defined feasibility as at least 50% of participants logging in for 30 out of the first 90 days. Most studies did not define feasibility but assessed metrics that we considered as feasibility. The majority (17/24, 71%) reported participant flow data from which enrollment, attrition, exclusion, or retention rates could be inferred [24-26,29,32-35,37,38,40-47]. Many studies (17/24, 71%) examined user-data metrics to assess intervention adherence [33,37,41], engagement [27,29,34,35,40,46], adoption [43], activation [35], intended use [46], and the extent of use [26,31,34,35,40,46]. “Intervention adherence” was assessed by conversation replies [33] and self-monitoring uploads [37]. Measures of “engagement” varied widely; for instance, one study [29] defined “engaged sessions” as the number of completed sessions, while other studies considered the quality of data reported by the participants [40] or the percentage of answered conversational turns [35]. One study [37] studied a similar measure, the ratio of conversations answered by the user and all conversations the CA initiated, rendering this as intervention adherence. Similarly, “extent of use” was measured inconsistently, varying from the number of consultations [31,35] to time spent in-app [46]. Some used “extent of use” to assess “intervention exposure” [26] and “received dosage” [38]. Others included use metrics without further specification of what they intended to measure [26,31,35,45] or reported what they measured without describing how [41]. In addition, 8.3% (2/24) of the studies did not evaluate feasibility [28,36].

#### Acceptability Outcomes

Some (10/24, 42%) studies reported acceptability. Acceptability was assessed with nonstandardized surveys [34,39,45], the extent of app use [45], usability using the System Usability Scale [32], or intervention satisfaction by a usability and acceptability survey combined with an interview to collect user experience and feedback [30]. One study [46] reported “engagement and acceptance,” including intention and commitment to change behavior, working alliance, participants’ sensitivity to triggers, and tendency to avoid triggers. Another study [44] reported on “patient use and acceptance of CAs,” encompassing use of CA (tools and reminders), acceptance (#users that did not uninstall signal), and usefulness (#times the patient was not understood by the CA). One study [25] defined an acceptability threshold as 50% of patients agreeing to participate. Some of these studies included additional metrics that we classified as acceptability, such as usability [25] and working alliance [33] using standardized questionnaires, helpfulness of the intervention [25], and perceived enjoyment using one single question [46].

Some authors did not report on acceptability but reported measures that we classified as acceptability, such as usability assessed with standardized questionnaires (ie, Chatbot Usability Questionnaire [28] or System Usability Scale [40,47,48]), CA feedback [29], helpfulness of the intervention [29], or user experience and satisfaction [35,36,40] using self-compromised surveys consisting of (single) questions, ratings, or the User Experience Questionnaire [40]. In addition, 13% (3/24) of studies interviewed their participants about the user experience [30,42,44]. One study [31] referred to patient satisfaction in their results, but the method description lacked, and another reported measures related to user experience but left them undefined [42]. The client-satisfaction questionnaire was used to address attitudes toward the intervention in one study [47] and usability in another [24,25].

### Primary or Coprimary Study Results and RoB

Most study findings support the effectiveness of the CAs [28,31,34,38,41,46,48]. Their interventions were effective in elevating (health-related) QoL in patients with atrial fibrillation [41], diabetes [43], inflammatory bowel syndrome [38], and overactive bladder [48]. Other studies showed that their interventions improved mental well-being in patients with chronic headaches [46] and diminished symptom severity in patients with inflammatory bowel syndrome [38]. In cancer, the interventions led to fewer emergency department visits of patients with gynecologic cancer [31] and diminished chemotherapy-related side effects and improved effectiveness of self-care behaviors in breast cancer [28]. In diabetes, 8% (2/24) of studies found improved blood glucose levels [34,35] while another study found no effect on blood glucose levels [43]. Pain-related impairment in chronic pain did not differ between the intervention and control group [33]. Another CA intervention did not significantly improve medication adherence in young men who were HIV positive and have sex with men [45]. Furthermore, one study showed acceptability of their CA with a high overall user satisfaction (1/24, 4%) [39].

All included studies that reported primary outcomes were rated as having a high, serious, or critical RoB (Multimedia Appendix 2 [28,31,33-35,38,41,43,45,46,48]. Missing outcome data were a shared concern in randomized controlled trials and nonrandomized trials: 29% (7/24) of studies reported substantial missing data without providing reasons or using appropriate methods to account for the missing data [33-35,38,43,45,46]. In the randomized controlled trials, the most common source of high RoB was in outcome measurement [28,33,38,41,43,46]. Given the nature of the interventions, participants were often unblinded, and primary outcomes—such as QoL—were self-reported and susceptible to influence by participants’ expectations or beliefs about the intervention. In nonrandomized trials, bias also often emerged from the lack of controlling for key confounding factors, such as age, digital skills, comorbidities, and disease stage [34,35,45,48].

## Discussion

### Principal Findings

This review provides a comprehensive overview of the current state of intervention descriptions and evaluation methods for CAs supporting people to self-manage chronic diseases. Despite promising development, significant gaps remain in intervention descriptions and evaluations. We discuss these findings in further detail, highlighting existing strengths and directions for future research.

### Characteristics of CAs and Integration of BCTs

Consistent with prior reviews [11,14], nearly all CAs were designed for specific diseases, such as cancer and diabetes. This allows CAs to be designed attuned to the disease-specific needs of people. Interventions aimed at improving adherence, self-care confidence, and behavior. However, intervention descriptions were inconsistent or incomplete, particularly conversational techniques, hindering understanding how responses are generated and insights into conversational flows, limiting replicability. To illustrate, one study reported about AI-powered decision support [34], but because the description of the conversational techniques is lacking, it is unclear how this functionality works. This aligns with earlier research highlighting inconsistent documentation of AI methodologies in CAs for chronic disease self-management [11]. Despite the rapid advancements in AI, the number of AI-driven CAs identified in our selection was lower than expected, and many AI-driven CAs relied on basic natural language processing rather than more advanced AI capabilities. This highlights the early-stage nature of AI integration in health care CA research. This is further supported by our observation that studies investigating more advanced AI-driven CAs evaluated for response accuracy rather than their effects on disease self-management.

BCT clusters “feedback and monitoring,” “shaping knowledge,” “natural consequences,” and “associations” were most prominently integrated. Findings from other studies examining eHealth interventions underscore the prevalence of feedback and monitoring, shaping knowledge, and associations [51,52]. “Associations” were often prompts to CA use, which can also be considered a persuasive feature of technology. Furthermore, CAs demonstrate the unique capacity to deliver “social support” by serving as a source of support themselves. Explicit reporting on BCT integration is often lacking. Only a few studies explicitly reported the theoretical frameworks guiding their interventions or the BCTs used, a concern echoed by a previous review on BCTs in self-management interventions for chronic obstructive pulmonary disease [53]. This raises the question of whether the intervention was designed using established theoretical frameworks or if such frameworks were absent. The latter could potentially result in less effective interventions. Conversely, if a theoretical basis exists, insufficient reporting generates ambiguity in two critical areas: (1) the chosen BCTs and (2) the role of technology in delivering these techniques. Clear reporting of the BCTs and their delivery—such as Ulrich et al [46]—is crucial to evaluate the theoretical underpinnings, providing insights into the mechanism behind behavior change and improving replicability and comparability.

### Methodological Limitations in Intervention Evaluation

The primary findings of the studies supported the potential of CAs to improve self-management and health outcomes. A previous review similarly suggested that AI-powered chatbots may contribute to better health outcomes; however, the evidence was limited due to insufficient technical documentation [12]. These findings should be interpreted with caution, as all included studies exhibited a heightened RoB—a concern raised by earlier literature as well [12].

The field of CA interventions for chronic disease self-management remains in an early stage [11,14], with many studies being exploratory. Also, the heterogeneity in study designs, variability in taxonomy, and use of broad (not unified) outcomes present a major challenge [11,14,18]. Consequently, the possibility of conducting a meta-analysis is prevented [12,47]. Many studies relied on nonstandardized acceptability surveys. Clinical outcomes were often self-reported. Although self-reported clinical outcomes are valuable for capturing patient experiences, they are also prone to subjectivity, recall, and social desirability biases [6]. Combining self-reported outcomes with objective measures, such as blood glucose levels [34,44], offers a more comprehensive perspective on the patient’s health, reduces bias, and enhances reliability. Another issue is that few studies measured outcomes related to self-management (eg, knowledge), despite their critical role in improving health, highlighting a significant gap we should overcome to understand how CAs contribute to improving health outcomes.

Selection bias is a concern. Participants were often required to possess a compatible device. This eligibility criterion excludes less technology-oriented individuals or individuals with lower income, thereby limiting the generalizability of findings to more diverse populations. Dworkin et al [45] tried to overcome this bias by providing a study loaner phone. Moreover, minimal attention was given to patients’ technical proficiency, a factor that directly impacts engagement and outcomes, complicating the understanding of whether limited effects are due to the intervention or users’ technological comfort. Recommended would be at least an approach that describes the technological proficiency of the study group using standardized questionnaires [48].

While CAs are often proposed as solutions to health care resource scarcity, studies provided minimal details about the care environment where the interventions were implemented, and it was often unclear whether they opted to supplement or replace usual care. Also, the associated time investment of human involvement was not reported. Both hamper the understanding of the resource implication of implementing these interventions.

### Limitations

This study was not without shortcomings. Only 2 databases were used in our search. Consequently, we might have missed some potentially relevant articles. However, our search in Embase and PubMed gives a good presentation of the articles published within the biomedical field—which was our key focus. Also, we included a considerable number of papers, and our main findings were consistently observed throughout the articles included. Therefore, even if some articles were missing, it is unlikely that additional articles would alter our results. The article inclusion and data extraction process were conducted by a single researcher, which increases the risk of subjective bias. To mitigate this, the inclusion and selection process was frequently discussed and reviewed with 2 additional researchers whenever uncertainties about an article’s inclusion arose, and the extracted data was cross-checked with the original articles to ensure accuracy. Moreover, the categorization of BCT clusters involved a degree of interpretation, which could affect the consistency of the findings. However, by categorizing BCTs in consensus meetings with 3 researchers, we minimized this risk. The limited detail in intervention reporting may have led to an underestimation of the presence and diversity of BCTs in this analysis. This concern is supported by the observation that the only study [46] that reported what and how BCTs were delivered included the most (diverse) BCT clusters. The same applies to the classification of outcome variables and conversational techniques, which require subjective judgment. However, to enable comparability across studies, this categorization was the preferred option.

### Recommendations for Future CAs in Health Care Research

To improve the rapidly evolving field of research on CA for chronic disease self-management, our review highlights several areas for advancing the evaluation of CAs.

#### Standardize Reporting

Adopt frameworks like CONSORT-eHealth to ensure comprehensive documentation of the study and the BIT model for detailed reports of CA intervention components, separating between BCTs and their delivery by technology. We believe this enhances replicability and comparability across studies and allows us to evaluate what (combinations of) BCTs are most effective for chronic disease self-management and how they should be delivered to the user. This contributes to more robust and transparent evidence, including a better understanding of the working mechanisms of CAs as self-management interventions. If a study aims to further characterize the dialogue management systems of CAs, the framework by Laranjo et al [54] offers a valuable basis to refine CA characterization and technical evaluation.

#### Enhance and Broaden Methodological Rigor

Use validated, standardized evaluation methods for feasibility, acceptability, and clinical efficacy. Also, add objective disease-specific and technology (eg, log data for reach and engagement) measures alongside self-reported outcomes to strengthen the evidence of clinical effectiveness and adoption of the CA. Objective measures are essential to minimize bias, given the challenges of blinding participants in CA interventions. The use of shared taxonomy and clear definitions about the intended measure are key. This will improve reliability and comparability across studies and allow meta-analysis across multiple studies. As attrition is typically high in eHealth trials [21,55], including those in our assessment, robust strategies for handling missing data—such as sensitivity analysis [23], multiple imputation [56] using relevant covariates, and transparent reporting of missingness and its causes [23]—are essential to reduce bias. In addition, greater attention should be given to key factors, such as age, digital skills, health or eHealth literacy, comorbidities, and disease stage, which may influence CA engagement and effectiveness. Incorporate methodological frameworks of multiple domains, such as human-centered design research, to further CA research into a more agile field of science [57].

#### Conduct Real-World Evaluations

This includes evaluating the role of CAs in supplementing or replacing traditional care, reporting on associated resource implications, and examining their impact on health care use. We recommend evaluating the implementation of CAs for self-management using established, well-defined outcomes with respect to BIT use in routine practice settings [58] and using real-world evaluation frameworks that consider the complexity of the health care system as an evaluation context [59,60].

#### Leverage Advanced AI

Expanding the use of AI-driven personalization and decision-support functionalities may enhance user engagement and intervention efficacy. Improved personalization based on individual data and dynamic adapting and learning from the interaction can support self-management by fostering better decision-making (eg, predicting behavioral patterns and identifying potential challenges and proactively suggest solutions) and self-tailoring (refining suggestions and ensuring interventions remain relevant to the user). Furthermore, incorporating sentiment analysis into CAs enhances their capacity to deliver social support that is both empathetic and contextually appropriate [18].

## Appendix

Multimedia Appendix 1 PRISMA (Preferred Reporting Items for Systematic Reviews and Meta-Analyses) 2020 checklist.

Multimedia Appendix 2 Risk of bias assessment.

## Abbreviations

AI: artificial intelligence
BCT: behavior change technique
BIT: behavioral intervention technology
CA: conversational agent
CONSORT: Consolidated Standards of Reporting Trials
LLM: large language model
PRISMA: Preferred Reporting Items for Systematic Reviews and Meta-Analyses
QoL: quality of life
ROBINS-I: Risk of Bias in Non-randomized Studies-of Intervention

## Data Availability

The data that support the findings of this study are available from the corresponding author upon reasonable request.

## References

[1] Vandenberghe D, Albrecht J (2020). The financial burden of non-communicable diseases in the European Union: a systematic review. Eur J Public Health.

[2] Hacker K (2024). The burden of chronic disease. Mayo Clin Proc Innov Qual Outcomes.

[3] Grady PA, Gough LL (2014). Self-management: a comprehensive approach to management of chronic conditions. Am J Public Health.

[4] Allegrante JP, Wells MT, Peterson JC (2019). Interventions to support behavioral self-management of chronic diseases. Annu Rev Public Health.

[5] Lorig KR, Holman HR (2003). Self-management education: history, definition, outcomes, and mechanisms. Ann Behav Med.

[6] Griffin AC, Xing Z, Khairat S, Wang Y, Bailey S, Arguello J, Chung AE (2020). Conversational agents for chronic disease self-management: a systematic review. AMIA Annu Symp Proc.

[7] Barlow J, Wright C, Sheasby J, Turner A, Hainsworth J (2002). Self-management approaches for people with chronic conditions: a review. Patient Educ Couns.

[8] Romano MF, Shih LC, Paschalidis IC, Au R, Kolachalama VB (2023). Large language models in neurology research and future practice. Neurology.

[9] Michie S, Richardson M, Johnston M, Abraham C, Francis J, Hardeman W, Eccles MP, Cane J, Wood CE (2013). The behavior change technique taxonomy (v1) of 93 hierarchically clustered techniques: building an international consensus for the reporting of behavior change interventions. Ann Behav Med.

[10] Mohr DC, Schueller SM, Montague E, Burns MN, Rashidi P (2014). The behavioral intervention technology model: an integrated conceptual and technological framework for eHealth and mHealth interventions. J Med Internet Res.

[11] Bin Sawad A, Narayan B, Alnefaie A, Maqbool A, Mckie I, Smith J, Yuksel B, Puthal D, Prasad M, Kocaballi AB (2022). A systematic review on healthcare artificial intelligent conversational agents for chronic conditions. Sensors (Basel).

[12] Kurniawan MH, Handiyani H, Nuraini T, Hariyati RTS, Sutrisno S (2024). A systematic review of artificial intelligence-powered (AI-powered) chatbot intervention for managing chronic illness. Ann Med.

[13] Moura L, Jones DT, Sheikh IS, Murphy S, Kalfin M, Kummer BR, Weathers AL, Grinspan ZM, Silsbee HM, Jones LK, Patel AD (2024). Implications of large language models for quality and efficiency of neurologic care: emerging issues in neurology. Neurology.

[14] Schachner T, Keller R, V Wangenheim F (2020). Artificial intelligence-based conversational agents for chronic conditions: systematic literature review. J Med Internet Res.

[15] Patrick K, Hekler EB, Estrin D, Mohr DC, Riper H, Crane D, Godino J, Riley WT (2016). The pace of technologic change: implications for digital health behavior intervention research. Am J Prev Med.

[16] Bérubé C, Schachner T, Keller R, Fleisch E, V Wangenheim F, Barata F, Kowatsch T (2021). Voice-based conversational agents for the prevention and management of chronic and mental health conditions: systematic literature review. J Med Internet Res.

[17] Jiang Z, Huang X, Wang Z, Liu Y, Huang L, Luo X (2024). Embodied conversational agents for chronic diseases: scoping review. J Med Internet Res.

[18] Uetova E, Hederman L, Ross R, O'Sullivan D (2024). Exploring the characteristics of conversational agents in chronic disease management interventions: a scoping review. Digit Health.

[19] Page MJ, McKenzie JE, Bossuyt PM, Boutron I, Hoffmann TC, Mulrow CD, Shamseer L, Tetzlaff JM, Akl EA, Brennan SE, Chou R, Glanville J, Grimshaw JM, Hróbjartsson A, Lalu MM, Li T, Loder EW, Mayo-Wilson E, McDonald S, McGuinness LA, Stewart LA, Thomas J, Tricco AC, Welch VA, Whiting P, Moher D (2021). The PRISMA 2020 statement: an updated guideline for reporting systematic reviews. BMJ.

[20] About chronic diseases. Centers for Disease Control and Prevention.

[21] Eysenbach G, CONSORT-EHEALTH Group (2011). CONSORT-EHEALTH: improving and standardizing evaluation reports of web-based and mobile health interventions. J Med Internet Res.

[22] Sterne JA, Hernán MA, Reeves BC, Savović J, Berkman ND, Viswanathan M, Henry D, Altman DG, Ansari MT, Boutron I, Carpenter JR, Chan AW, Churchill R, Deeks JJ, Hróbjartsson A, Kirkham J, Jüni P, Loke YK, Pigott TD, Ramsay CR, Regidor D, Rothstein HR, Sandhu L, Santaguida PL, Schünemann HJ, Shea B, Shrier I, Tugwell P, Turner L, Valentine JC, Waddington H, Waters E, Wells GA, Whiting PF, Higgins JP (2016). ROBINS-I: a tool for assessing risk of bias in non-randomised studies of interventions. BMJ.

[23] Sterne JA, Savović J, Page MJ, Elbers RG, Blencowe NS, Boutron I, Cates CJ, Cheng H, Corbett MS, Eldridge SM, Emberson JR, Hernán MA, Hopewell S, Hróbjartsson A, Junqueira DR, Jüni P, Kirkham JJ, Lasserson T, Li T, McAleenan A, Reeves BC, Shepperd S, Shrier I, Stewart LA, Tilling K, White IR, Whiting PF, Higgins JP (2019). RoB 2: a revised tool for assessing risk of bias in randomised trials. BMJ.

[24] Caru M, Abdullah S, Qiu L, Kanski B, Gordon B, Truica CI, Vasakar M, Doerksen S, Schmitz KH (2023). Women with metastatic breast cancer don't just follow step-count trends, they exceed them: an exploratory study. Breast Cancer Res Treat.

[25] Schmitz KH, Kanski B, Gordon B, Caru M, Vasakar M, Truica CI, Wang M, Doerksen S, Lorenzo A, Winkels R, Qiu L, Abdullah S (2023). Technology-based supportive care for metastatic breast cancer patients. Support Care Cancer.

[26] Cheng AL, Agarwal M, Armbrecht MA, Abraham J, Calfee RP, Goss CW (2023). Behavioral mechanisms that mediate mental and physical health improvements in people with chronic pain who receive a digital health intervention: prospective cohort pilot study. JMIR Form Res.

[27] Meheli S, Sinha C, Kadaba M (2022). Understanding people with chronic pain who use a cognitive behavioral therapy-based artificial intelligence mental health app (Wysa): mixed methods retrospective observational study. JMIR Hum Factors.

[28] Tawfik E, Ghallab E, Moustafa A (2023). A nurse versus a chatbot ‒ the effect of an empowerment program on chemotherapy-related side effects and the self-care behaviors of women living with breast Cancer: a randomized controlled trial. BMC Nurs.

[29] Greer S, Ramo D, Chang Y, Fu M, Moskowitz J, Haritatos J (2019). Use of the chatbot "Vivibot" to deliver positive psychology skills and promote well-being among young people after cancer treatment: randomized controlled feasibility trial. JMIR Mhealth Uhealth.

[30] Gomaa S, Posey J, Bashir B, Basu Mallick A, Vanderklok E, Schnoll M, Zhan T, Wen K (2023). Feasibility of a text messaging-integrated and chatbot-interfaced self-management program for symptom control in patients with gastrointestinal cancer undergoing chemotherapy: pilot mixed methods study. JMIR Form Res.

[31] Huang MY, Weng CS, Kuo HL, Su YC (2023). Using a chatbot to reduce emergency department visits and unscheduled hospitalizations among patients with gynecologic malignancies during chemotherapy: a retrospective cohort study. Heliyon.

[32] Chen NJ, Huang C, Fan C, Lu L, Lin F, Liao J, Guo J (2023). User evaluation of a chat-based instant messaging support health education program for patients with chronic kidney disease: preliminary findings of a formative study. JMIR Form Res.

[33] Hauser-Ulrich S, Künzli H, Meier-Peterhans D, Kowatsch T (2020). A smartphone-based health care chatbot to promote self-management of chronic pain (SELMA): pilot randomized controlled trial. JMIR Mhealth Uhealth.

[34] Krishnakumar A, Verma R, Chawla R, Sosale A, Saboo B, Joshi S, Shaikh M, Shah A, Kolwankar S, Mattoo V (2021). Evaluating glycemic control in patients of south Asian origin with type 2 diabetes using a digital therapeutic platform: analysis of real-world data. J Med Internet Res.

[35] Nassar CM, Dunlea R, Montero A, Tweedt A, Magee MF (2023). Feasibility and preliminary behavioral and clinical efficacy of a diabetes education chatbot pilot among adults with type 2 diabetes. J Diabetes Sci Technol.

[36] Echeazarra L, Pereira J, Saracho R (2021). TensioBot: a chatbot assistant for self-managed in-house blood pressure checking. J Med Syst.

[37] Sakane N, Suganuma A, Domichi M, Sukino S, Abe K, Fujisaki A, Kanazawa A, Sugimoto M (2023). The effect of a mHealth app (KENPO-app) for specific health guidance on weight changes in adults with obesity and hypertension: pilot randomized controlled trial. JMIR Mhealth Uhealth.

[38] Hunt M, Miguez S, Dukas B, Onwude O, White S (2021). Efficacy of Zemedy, a mobile digital therapeutic for the self-management of irritable bowel syndrome: crossover randomized controlled trial. JMIR Mhealth Uhealth.

[39] Cheng CI, Lin WJ, Liu HT, Chen YT, Chiang CK, Hung KY (2023). Implementation of artificial intelligence Chatbot in peritoneal dialysis nursing care: experience from a Taiwan medical center. Nephrology (Carlton).

[40] Albino de Queiroz D, Silva Passarello R, Veloso de Moura Fé V, Rossini A, Folchini da Silveira E, Aparecida Isquierdo Fonseca de Queiroz E, André da Costa C (2023). A wearable chatbot-based model for monitoring colorectal cancer patients in the active phase of treatment. Healthc Anal.

[41] Guhl E, Althouse AD, Pusateri AM, Kimani E, Paasche-Orlow MK, Bickmore TW, Magnani JW (2020). The atrial fibrillation health literacy information technology trial: pilot trial of a mobile health app for atrial fibrillation. JMIR Cardio.

[42] Fang KY, Bjering H, Ginige A (2018). Adherence, avatars and where to from here. Stud Health Technol Inform.

[43] Gong E, Baptista S, Russell A, Scuffham P, Riddell M, Speight J, Bird D, Williams E, Lotfaliany M, Oldenburg B (2020). My diabetes coach, a mobile app-based interactive conversational agent to support type 2 diabetes self-management: randomized effectiveness-implementation trial. J Med Internet Res.

[44] Roca S, Lozano ML, García José, Alesanco ? (2021). Validation of a virtual assistant for improving medication adherence in patients with comorbid type 2 diabetes mellitus and depressive disorder. Int J Environ Res Public Health.

[45] Dworkin MS, Lee S, Chakraborty A, Monahan C, Hightow-Weidman L, Garofalo R, Qato DM, Liu L, Jimenez A (2019). Acceptability, feasibility, and preliminary efficacy of a theory-based relational embodied conversational agent mobile phone intervention to promote HIV medication adherence in young HIV-positive African American MSM. AIDS Educ Prev.

[46] Ulrich S, Gantenbein AR, Zuber V, Von Wyl A, Kowatsch T, Künzli H (2024). Development and evaluation of a smartphone-based chatbot coach to facilitate a balanced lifestyle in individuals with headaches (BalanceUP app): randomized controlled trial. J Med Internet Res.

[47] Bruijnes M, Kesteloo M, Brinkman WP (2023). Reducing social diabetes distress with a conversational agent support system: a three-week technology feasibility evaluation. Front Digit Health.

[48] Sheyn D, Chakraborty N, Chen YB, Mahajan ST, Hijaz A (2024). Use of a digital conversational agent for the management of overactive bladder. Urogynecology (Phila).

[49] Qiu L, Kanski B, Doerksen S, Winkels R, Schmitz KH, Abdullah S (2021). Nurse AMIE: using smart speakers to provide supportive care intervention for women with metastatic breast cancer. Proceedings of the 2021 CHI Conference on Human Factors in Computing Systems.

[50] Roca S, Sancho J, García J, Alesanco Á (2020). Microservice chatbot architecture for chronic patient support. J Biomed Inform.

[51] Martinengo L, Jabir AI, Goh WW, Lo NY, Ho MR, Kowatsch T, Atun R, Michie S, Tudor Car L (2022). Conversational agents in health care: scoping review of their behavior change techniques and underpinning theory. J Med Internet Res.

[52] Asbjørnsen RA, Smedsrød ML, Solberg Nes L, Wentzel J, Varsi C, Hjelmesæth J, van Gemert-Pijnen JE (2019). Persuasive system design principles and behavior change techniques to stimulate motivation and adherence in electronic health interventions to support weight loss maintenance: scoping review. J Med Internet Res.

[53] Lenferink A, Brusse-Keizer M, van der Valk PD, Frith PA, Zwerink M, Monninkhof EM, van der Palen J, Effing TW (2017). Self-management interventions including action plans for exacerbations versus usual care in patients with chronic obstructive pulmonary disease. Cochrane Database Syst Rev.

[54] Laranjo L, Dunn AG, Tong HL, Kocaballi AB, Chen J, Bashir R, Surian D, Gallego B, Magrabi F, Lau AY, Coiera E (2018). Conversational agents in healthcare: a systematic review. J Am Med Inform Assoc.

[55] Eysenbach G (2005). The law of attrition. J Med Internet Res.

[56] Blankers M, Koeter MW, Schippers GM (2010). Missing data approaches in eHealth research: simulation study and a tutorial for nonmathematically inclined researchers. J Med Internet Res.

[57] Hekler EB, Klasnja P, Harlow J, Gellman MD (2020). Agile science. Encyclopedia of Behavioral Medicine.

[58] Hermes ED, Lyon AR, Schueller SM, Glass JE (2019). Measuring the implementation of behavioral intervention technologies: recharacterization of established outcomes. J Med Internet Res.

[59] Kim M, Patrick K, Nebeker C, Godino J, Stein S, Klasnja P, Perski O, Viglione C, Coleman A, Hekler E (2024). The digital therapeutics real-world evidence framework: an approach for guiding evidence-based digital therapeutics design, development, testing, and monitoring. J Med Internet Res.

[60] Greenhalgh T, Abimbola S (2019). The NASSS framework - a synthesis of multiple theories of technology implementation. Stud Health Technol Inform.

