# Supplementary material for: Conversational Agents Supporting Self-Management in People With a Chronic Disease: Systematic Review
Source: J Med Internet Res. 2025 Aug 26;27:e72309. doi: 10.2196/72309 (PMC12421203; doi:10.2196/72309)
Supplement: Multimedia Appendix 2 [file jmir_v27i1e72309_app2.pdf]

Multimedia appendix 2: Risk of bias assessment

Supplementary figure 1. Risk of bias assessment using Risk of Bias 2.0 Tool [1] for randomized controlled trials. One cross-over randomized trial [2] was assessed using the Risk of Bias 2.0 tool for cross-over trials. The primary outcome compared the intervention and waitlist control group before the waitlist group started with the intervention. Thus, the crossover-specific domain was not relevant to the outcome assessed. Consequently, we present this study [2] alongside the other randomized controlled trials in the same figure.

Study	D1	D2	D3	D4	D5	Total
Guhl et al [3], health-related QoL[a]	−	+	+	×	−	×
Tawfik et al [4], frequency, severity and distress of physical and psychological chemotherapy-related side effects	+	+	+	×	−	×
Tawfik et al [4], effectiveness of self-care behavior	+	+	+	×	−	×
Hauser-Ulrich et al [5], pain-related impairment	+	+	×	×	×	×
Gong et al [6], Hb1Ac[b]	+	+	×	+	×	×
Gong et al [6], health-related QoL	−	+	×	×	×	×
Ulrich et al [7], mental wellbeing	+	+	×	×	−	×
Hunt et al [2], QoL	−	+	×	×	−	×
Hunt et al [2], Symptom severity	+	+	×	×	−	×

Domains:
D1: Bias arising from the randomization process.
D2: Bias due to deviations from the intended intervention.
D3: Bias due to missing outcome data.
D4: Bias in measurement of the outcome.
D5: Bias in selection of the reported restult.

Judgement
× High
− Some concerns
\+ Low

[a]QoL: Quality of life
[b]Hb1Ac: Hemoglobin A1c

Supplementary figure 2. Risk of bias assessment using Risk of Bias in Non-randomized Studies-of Intervention-I tool [8] version 2 for non-randomized trials.

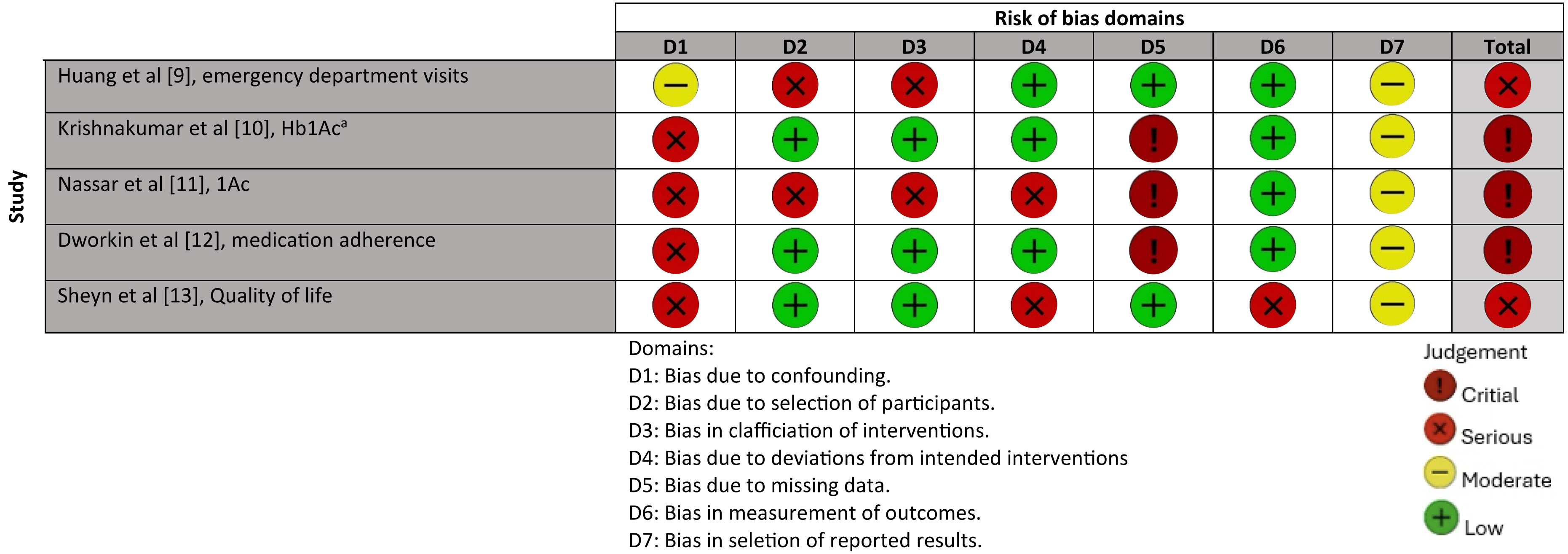

[a]Hb1Ac: Hemoglobin A1c

Reference list

[1] J. A. C. Sterne *et al.*, "RoB 2: a revised tool for assessing risk of bias in randomised trials," (in English), *Bmj-Brit Med J,* vol. 366, Aug 28 2019, doi: ARTN l4898

10.1136/bmj.l4898.PMID: WOS:000483891600002

[2] M. Hunt, S. Miguez, B. Dukas, O. Onwude, and S. White, "Efficacy of Zemedy, a Mobile Digital Therapeutic for the Self-management of Irritable Bowel Syndrome: Crossover Randomized Controlled Trial," *JMIR Mhealth Uhealth,* vol. 9, no. 5, p. e26152, May 20 2021, doi: 10.2196/26152.PMID: 33872182

[3] E. Guhl *et al.*, "The Atrial Fibrillation Health Literacy Information Technology Trial: Pilot Trial of a Mobile Health App for Atrial Fibrillation," *JMIR Cardio,* vol. 4, no. 1, p. e17162, Sep 4 2020, doi: 10.2196/17162.PMID: 32886070

[4] E. Tawfik, E. Ghallab, and A. Moustafa, "A nurse versus a chatbot – the effect of an empowerment program on chemotherapy-related side effects and the self-care behaviors of women living with breast Cancer: a randomized controlled trial," *BMC Nurs,* vol. 22, no. 1, p. 102, Apr 6 2023, doi: 10.1186/s12912-023-01243-7.PMID: 37024875

[5] S. Hauser-Ulrich, H. Kunzli, D. Meier-Peterhans, and T. Kowatsch, "A Smartphone-Based Health Care Chatbot to Promote Self-Management of Chronic Pain (SELMA): Pilot Randomized Controlled Trial," *JMIR Mhealth Uhealth,* vol. 8, no. 4, p. e15806, Apr 3 2020, doi: 10.2196/15806.PMID: 32242820

[6] E. Gong *et al.*, "My Diabetes Coach, a Mobile App-Based Interactive Conversational Agent to Support Type 2 Diabetes Self-Management: Randomized Effectiveness-Implementation Trial," *J Med Internet Res,* vol. 22, no. 11, p. e20322, Nov 5 2020, doi: 10.2196/20322.PMID: 33151154

[7] S. Ulrich, A. R. Gantenbein, V. Zuber, A. Von Wyl, T. Kowatsch, and H. Kunzli, "Development and Evaluation of a Smartphone-Based Chatbot Coach to Facilitate a Balanced Lifestyle in Individuals With Headaches (BalanceUP App): Randomized Controlled Trial," *J Med Internet Res,* vol. 26, p. e50132, Jan 24 2024, doi: 10.2196/50132.PMID: 38265863

[8] J. A. Sterne *et al.*, "ROBINS-I: a tool for assessing risk of bias in non-randomised studies of interventions," (in eng), *Bmj,* vol. 355, p. i4919, Oct 12 2016, doi: 10.1136/bmj.i4919.PMID: 27733354

[9] M. Y. Huang, C. S. Weng, H. L. Kuo, and Y. C. Su, "Using a chatbot to reduce emergency department visits and unscheduled hospitalizations among patients with gynecologic malignancies during chemotherapy: A retrospective cohort study," *Heliyon,* vol. 9, no. 5, p. e15798, May 2023, doi: 10.1016/j.heliyon.2023.e15798.PMID: 37206031

[10] A. Krishnakumar *et al.*, "Evaluating Glycemic Control in Patients of South Asian Origin With Type 2 Diabetes Using a Digital Therapeutic Platform: Analysis of Real-World Data," *J Med Internet Res,* vol. 23, no. 3, p. e17908, Mar 25 2021, doi: 10.2196/17908.PMID: 33764306

[11] C. M. Nassar, R. Dunlea, A. Montero, A. Tweedt, and M. F. Magee, "Feasibility and Preliminary Behavioral and Clinical Efficacy of a Diabetes Education Chatbot Pilot Among Adults With Type 2 Diabetes," *J Diabetes Sci Technol,* p. 19322968231178020, Jun 6 2023, doi: 10.1177/19322968231178020.PMID: 37278191

[12] M. S. Dworkin *et al.*, "Acceptability, Feasibility, and Preliminary Efficacy of a Theory-Based Relational Embodied Conversational Agent Mobile Phone Intervention to Promote HIV Medication Adherence in Young HIV-Positive African American MSM," *AIDS Educ Prev,* vol. 31, no. 1, pp. 17-37, Feb 2019, doi: 10.1521/aeap.2019.31.1.17.PMID: 30742481

[13] D. Sheyn, N. Chakraborty, Y. B. Chen, S. T. Mahajan, and A. Hijaz, "Use of a Digital Conversational Agent for the Management of Overactive Bladder," *Urogynecology (Phila),* vol. 30, no. 6, pp. 536-544, Jun 1 2024, doi: 10.1097/SPV.0000000000001428.PMID: 37930265
